# Methodological Challenges in Randomized Controlled Trials of mHealth Interventions: Cross-Sectional Survey Study and Consensus-Based Recommendations

**DOI:** 10.2196/53187

**Published:** 2024-12-19

**Authors:** Jesus Lopez-Alcalde, L Susan Wieland, Yuqian Yan, Jürgen Barth, Mohammad Reza Khami, Siddharudha Shivalli, Cynthia Lokker, Harleen Kaur Rai, Paul Macharia, Sergi Yun, Elvira Lang, Agnes Bwanika Naggirinya, Concepción Campos-Asensio, Leila Ahmadian, Claudia M Witt

**Affiliations:** 1 Institute for Complementary and Integrative Medicine University Hospital Zurich Zurich Switzerland; 2 Faculty of Medicine Universidad Francisco de Vitoria Madrid Spain; 3 Instituto Ramón y Cajal de Investigación Sanitaria (IRYCIS), Unidad de Bioestadística Clínica Hospital Universitario Ramón y Cajal, Center for Biomedical Research in Epidemiology and Public Health Network (CIBERESP) Madrid Spain; 4 School of Medicine and Health Sciences George Washington University Washington, DC United States; 5 Research Center for Caries Prevention, Dentistry Research Institute Tehran University of Medical Sciences Tehran Iran; 6 Community Oral Health Department, School of Dentistry Tehran University of Medical Sciences Tehran Iran; 7 Department of Medical Statistics Faculty of Epidemiology and Public Health London School of Hygiene & Tropical Medicine London United Kingdom; 8 Department of Health Research Methods, Evidence, and Impact McMaster University Hamilton, ON Canada; 9 Digital Health and Wellness Research Group Department of Computer and Information Sciences University of Strathclyde Glasgow United Kingdom; 10 Department of Research and Programmes Kenyatta National Hospital Nairobi Kenya; 11 University of Nairobi Faculty of Health Sciences Nairobi Kenya; 12 Bio-Heart Cardiovascular Diseases Research Group Bellvitge Biomedical Research Institute L'Hospitalet de Llobregat Barcelona Spain; 13 Community Heart Failure Program Cardiology Department, Bellvitge University Hospital L’Hospitalet de Llobregat Barcelona Spain; 14 Internal Medicine Department Bellvitge University Hospital Barcelona Spain; 15 Center for Biomedical Research in Cardiovascular Diseases (CIBERCV) Instituto Salud Carlos III Madrid Spain; 16 Hypnalgesics, Comfort Talk Brookline, MA United States; 17 Infectious Diseases Institute, College of Health Sciences Makerere University Kampala Uganda; 18 Biblioteca Médica Hospital Universitario de Móstoles Móstoles Spain; 19 Fakher Mechatronic Research Center Kerman University of Medical Sciences Kerman Iran; 20 Institute for Complementary and Integrative Medicine University of Zurich Zurich Switzerland; 21 Institute for Social Medicine, Epidemiology and Health Economics Charité – Universitätsmedizin Berlin Berlin Germany

**Keywords:** digital health, eHealth, mobile health, mHealth, randomized controlled trial, survey, recommendations, intervention integrity, adherence, consensus, mobile phone

## Abstract

**Background:**

Mobile health (mHealth) refers to using mobile communication devices such as smartphones to support health, health care, and public health. mHealth interventions have their own nature and characteristics that distinguish them from traditional health care interventions, including drug interventions. Thus, randomized controlled trials (RCTs) of mHealth interventions present specific methodological challenges. Identifying and overcoming those challenges is essential to determine whether mHealth interventions improve health outcomes.

**Objective:**

We aimed to identify specific methodological challenges in RCTs testing mHealth interventions’ effects and develop consensus-based recommendations to address selected challenges.

**Methods:**

A 2-phase participatory research project was conducted. First, we sent a web-based survey to authors of mHealth RCTs. Survey respondents rated on a 5-point scale how challenging they found 21 methodological aspects in mHealth RCTs compared to non-mHealth RCTs. Nonsystematic searches until June 2022 informed the selection of the methodological challenges listed in the survey. Second, a subset of survey respondents participated in an online workshop to discuss recommendations to address selected methodological aspects identified in the survey. Finally, consensus-based recommendations were developed based on the workshop discussion and email interaction.

**Results:**

We contacted 1535 authors of mHealth intervention RCTs, of whom 80 (5.21%) completed the survey. Most respondents (74/80, 92%) identified at least one methodological aspect as more or much more challenging in mHealth RCTs. The aspects most frequently reported as more or much more challenging were those related to mHealth intervention integrity, that is, the degree to which the study intervention was implemented as intended, in particular managing low adherence to the mHealth intervention (43/77, 56%), defining adherence (39/79, 49%), measuring adherence (33/78, 42%), and determining which mHealth intervention components are used or received by the participant (31/75, 41%). Other challenges were also frequent, such as analyzing passive data (eg, data collected from smartphone sensors; 24/58, 41%) and verifying the participants’ identity during recruitment (28/68, 41%). In total, 11 survey respondents participated in the subsequent workshop (n=8, 73% had been involved in at least 2 mHealth RCTs). We developed 17 consensus-based recommendations related to the following four categories: (1) how to measure adherence to the mHealth intervention (7 recommendations), (2) defining adequate adherence (2 recommendations), (3) dealing with low adherence rates (3 recommendations), and (4) addressing mHealth intervention components (5 recommendations).

**Conclusions:**

RCTs of mHealth interventions have specific methodological challenges compared to those of non-mHealth interventions, particularly those related to intervention integrity. Following our recommendations for addressing these challenges can lead to more reliable assessments of the effects of mHealth interventions on health outcomes.

## Introduction

### Mobile Health: Concept and Potential Impact

Mobile health (mHealth) refers to using mobile communication devices, such as smartphones, tablets, or smartwatches, to support health, health care, and public health [1-3]. An mHealth app is software incorporated into a mobile device to improve health, health care services, and research [4]. Due to the pressure on health systems, there is an urgent need to invest in health and health systems via effective, feasible, and sustainable interventions [5,6]. mHealth has the potential to improve health and health care through different means. One example is apps that monitor users and collect information, such as exercise, heart rate, or medication adherence [7]. Other apps support health care providers in diagnostic procedures or clinical decision-making for treatments [8]. Finally, mHealth apps can also deliver health interventions, which is this study’s focus. Examples are apps to increase medication adherence or provide psychotherapeutic interventions (eg, cognitive behavioral therapies) to manage mental and physical health problems [3,9-14].

### Effectiveness of mHealth Interventions: Promising Results, but High-Quality Randomized Controlled Trials Are Still Needed

The mHealth market size is rapidly expanding. There are >350,000 health apps, and the global market will probably grow by 40% between 2022 and 2030 [15,16]. Developers often advertise mHealth apps as improving health and well-being, but sound research should support their claims on the effects of mHealth interventions on clinical outcomes. However, the evidence that mHealth interventions can improve health care processes, change patient behavior, and improve health outcomes is still uncertain [14,15,17]. Randomized controlled trials (RCTs) are considered the most rigorous method for evaluating the efficacy and effectiveness of health interventions because they minimize bias [18]. Therefore, RCTs are crucial for generating mHealth evidence and drawing firm conclusions about mHealth interventions’ effects [19-21].

### Specific Methodological Challenges of mHealth Intervention RCTs: A Potential Barrier for Rigorous mHealth Research

mHealth interventions have peculiarities that distinguish them from other health care interventions, such as drug interventions. For example, mHealth interventions are usually behavioral interventions that are more complex to define and standardize than pharmacological interventions [22]. In addition, evaluating and reporting mHealth intervention integrity (the degree to which the study intervention was implemented as intended) in RCTs can be challenging [23]. A recent systematic review by Tønning et al [24] outlines key methodological challenges in conducting RCTs for smartphone-based treatments in psychiatry. The challenges highlighted include the rapid pace of technological advancements, which may make interventions obsolete by the time articles are published. In addition, privacy and security issues often do not receive the attention they deserve. Moreover, mHealth RCTs lack consistency in how adherence to interventions is measured and how primary outcome measures are selected, making it difficult to compare results across different RCTs. Challenges related to missing data and the methods used for statistical analysis further complicate the assessment of mHealth interventions’ efficacy. To address these issues, Tønning et al [24] recommend adopting more flexible trial designs, giving priority to ethical considerations concerning privacy and security, using technology for improved adherence tracking, and following detailed protocols that align with the CONSORT-EHEALTH (Consolidated Standards of Reporting Trials of Electronic and Mobile Health Applications and Online Telehealth) guidelines [25], among other recommendations. Previous findings indicate that evaluating mHealth intervention effects via RCTs has specific methodological challenges. Our research is the first to solicit direct feedback from mHealth researchers on methodological challenges unique to mHealth RCTs.

A survey and a consensus exercise were chosen for this project to address the diverse methodological challenges in mHealth RCTs. Consensus methods allow for the integration of expert perspectives to produce recommendations. This approach is essential given the lack of consistent solutions to these challenges. The goal of the consensus exercise was to develop recommendations for researchers working in mHealth. The intended audience is global, with participation from experts across regions, making the recommendations relevant for both high- and low-resource settings.

Identifying and overcoming the methodological challenges specific to RCTs of mHealth interventions (from now on referred to as mHealth RCTs) can clarify whether mHealth improves health outcomes. This study had two aims: (1) to identify specific methodological challenges in RCTs evaluating the effects of mHealth interventions and (2) to develop consensus-based recommendations to address selected methodological challenges in mHealth RCTs.

## Methods

### Overview

**Figure 1:**
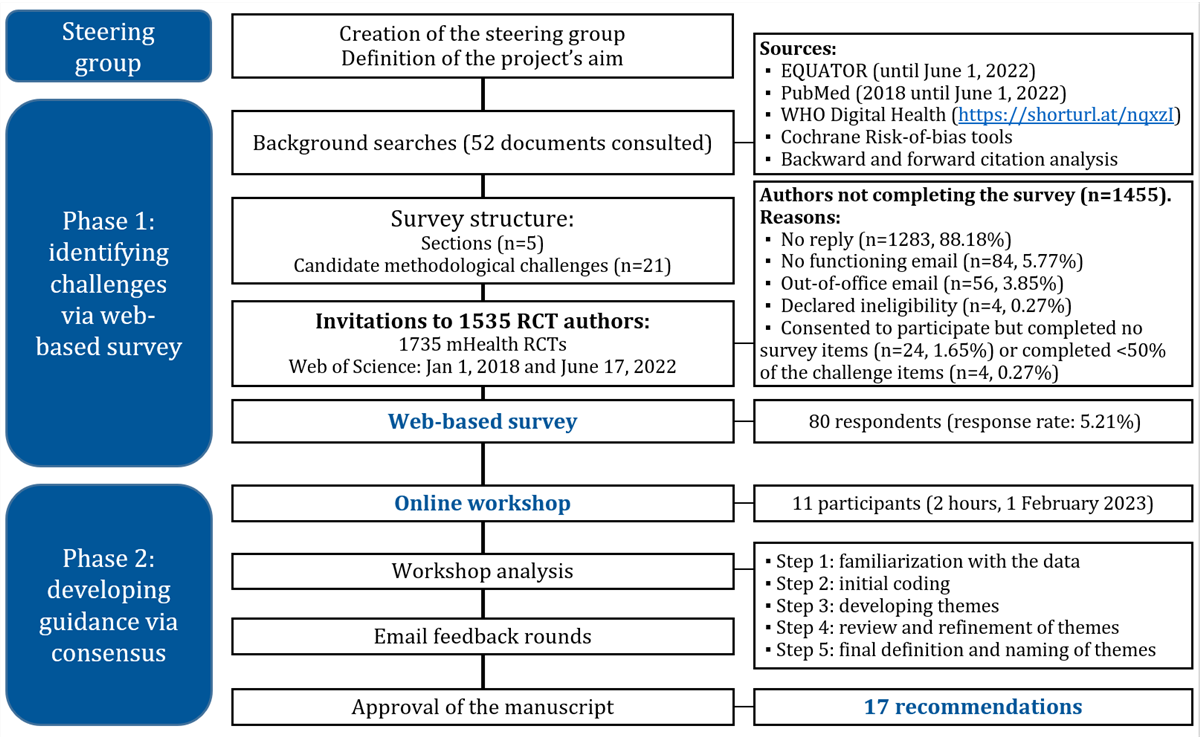
Study phases. EQUATOR: Enhancing the Quality and Transparency of Health Research; mHealth: mobile health; RCT: randomized controlled trial; WHO: World Health Organization.

We undertook a 2-phase participatory research study with mixed methods (quantitative and qualitative; Figure 1). This manuscript follows the Checklist for Reporting Results of Internet E-Surveys and the Accurate Consensus Reporting Document guidelines [26,27]. The study protocol was not registered.

The steering group, which included 4 researchers (CMW, LSW, JB, and JL-A), defined the project aims, collected potential methodological challenges of mHealth RCTs, identified the potential survey respondents, developed the survey, and selected the workshop participants. JB, an experienced researcher, facilitated the consensus meeting. The steering group also analyzed the survey results, summarized participants’ comments during the workshop, and integrated their feedback to generate this manuscript. No members of the public, patients, or carers were invited to participate in this study.

### Phase 1: Identifying Methodological Challenges Specific to mHealth RCTs

The study design was a cross-sectional web-based survey.

#### Survey Items

We created an anonymous open voluntary web-based survey in English using SoSci Survey (SoSci Survey GmbH)*.* The survey aimed at identifying methodological challenges specific to mHealth compared to non-mHealth RCTs. We performed nonsystematic searches in PubMed and EQUATOR (Enhancing the Quality and Transparency of Health Research) Network until June 1, 2022, looking for methodological challenges potentially relevant to mHealth RCTs (Figure 1; references of the documents consulted can be downloaded from Multimedia Appendix 1 [2,3,12,13,15,28-65]). On the basis of consensus, the steering group chose methodological aspects potentially specific to mHealth RCTs (initial list available upon request). The usability and technical functionality of the electronic questionnaire were tested by the steering group and 2 external researchers before fielding the questionnaire. The survey was available from September 23, 2022, to November 22, 2022 (Multimedia Appendix 2).

The first survey section listed 21 candidate methodological challenges in 5 sections (recruitment, randomization, intervention integrity, data quality, and data analysis). The questionnaire consisted of a total of 29 questions distributed over 6 pages with a maximum of 21 questions per page. The survey items were not randomized or alternated. Adaptive questioning (certain items were conditionally displayed based on responses to other items) was implemented to reduce the number of items. Participants described how challenging they found each methodological aspect in mHealth RCTs compared to non-mHealth RCTs. The response options were ranked on a 5-point scale: “much less challenging,” “less challenging,” “similar challenges,” “more challenging,” or “much more challenging.” An “I don’t know” option was also available. Participants could comment or propose additional challenges. Another survey section characterized the researchers’ academic background and experience with mHealth and fully remote RCTs (recruitment, delivery of the intervention, and evaluation were conducted online using mobile devices or the internet). After each section of the questionnaire was filled in, participants had the opportunity to check for completeness. If any items were left unanswered, a message would appear to highlight the missing responses. Completing the missing items was not mandatory to minimize survey fatigue. Respondents were able to review and change their answers by clicking on the back button. Finally, the survey invited the respondents to the online workshop (participation was optional, and responses were stored separately).

#### Survey Sample

The sample frame included a convenience sample of 1535 authors from 1735 mHealth RCTs indexed in Web of Science (January 1, 2018, to June 17, 2022) who were invited via email to complete the survey. One invitation email (September 23, 2022) and a reminder (October 14, 2022) outlined the survey’s purpose, duration, anonymity, funding source, the option to pause and resume the survey, and who the investigators were. Participants were encouraged to contact the team with questions. The email specified that the survey targeted trials evaluating mHealth interventions, excluding areas such as health monitoring or diagnosis. No incentives were offered to participate. Multimedia Appendix 2 provides the full invitation email. Participants could complete the survey multiple times as the survey platform did not restrict submissions to unique visitors. Cookies were not used to assign a unique user identifier to each client computer. In addition, IP addresses were not used to detect potential duplicate entries from the same user, and no other methods were used to analyze the log file for identifying multiple submissions.

A librarian (CC-A) designed the search strategy for identifying mHealth RCTs and obtaining the authors’ contact details (Multimedia Appendix 3). All survey respondents were required to confirm their participation in at least one mHealth RCT before completing the survey.

#### Survey Analysis

We used descriptive statistics (percentages, means and SDs, and medians and IQRs) to summarize dichotomous and quantitative data in narrative and tabular formats. We used R (R Foundation for Statistical Computing) to conduct statistical analyses [66]. We reported the proportion of participants who perceived each methodological aspect as more or much more challenging for mHealth compared to non-mHealth RCTs. Individuals who consented to participate but rated <50% of the listed challenges were excluded. Participants who responded “I don’t know” or did not respond to a particular item were excluded from the analysis for that item. There was no time limit for completing the questionnaire. The results are presented for the entire survey sample and stratified according to the researchers’ experience (experienced researchers were involved in more than one mHealth RCT). There were no statistical corrections to adjust for the nonrepresentative sample. Thematic analysis was used to group participants’ comments into overarching categories [67] according to a 5-step process. JL-A conducted steps 1 to 3 (familiarization with the data, initial coding, and developing themes according to the topics of the survey). CMW reviewed and refined the themes in step 4, and then JL-A and CMW defined and named the themes in step 5.

### Phase 2: Developing Consensus-Based Recommendations to Address Methodological Challenges in mHealth RCTs

The consensus method involved a consensus meeting, wherein participants engaged in discussions to reach an agreement. Recommendations from the workshop were endorsed through consensus without structured voting [26] (Multimedia Appendix 4).

#### Online Workshop

**Figure 2:**
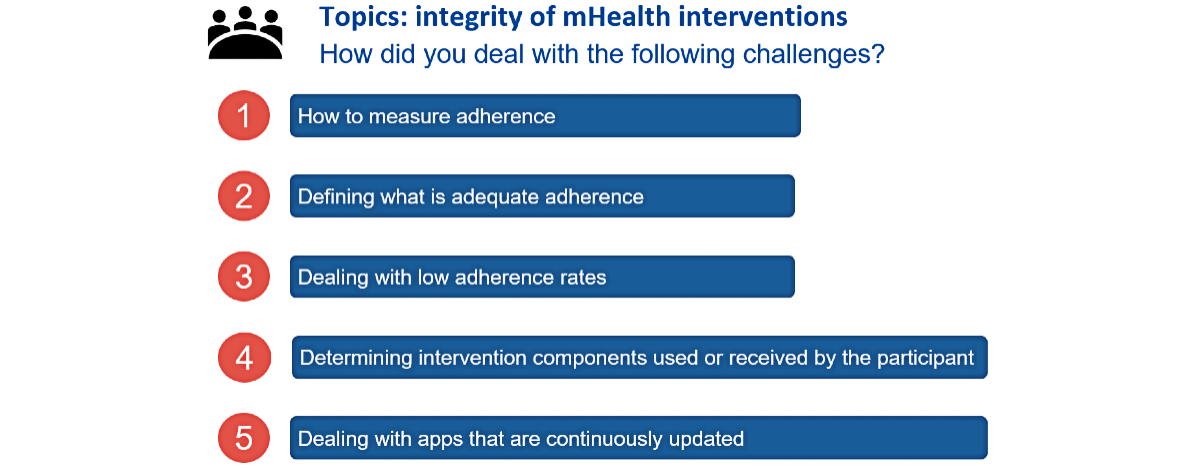
Workshop topics. mHealth: mobile health.

A 2-hour online workshop was held with mHealth researchers via a Zoom session (Zoom Video Communications) and was recorded with the participants’ consent. We invited the 52 survey respondents who indicated their interest in participating in a workshop to develop consensus-based recommendations addressing methodological challenges specific to mHealth RCTs. The workshop included 4 sessions: (1) introduction of the participants, (2) project aims and survey results, (3) development of recommendations to overcome methodological challenges in mHealth RCTs, and (4) closing remarks. The workshop focused on 2 topics: mHealth intervention integrity and dealing methodologically with continuously updated apps (Figure 2). The first topic was frequently rated as more or much more challenging by the experienced mHealth researchers in the survey. The steering group chose to add the second topic for 2 reasons. First, the continuous requirement to update the apps is also related to mHealth intervention integrity. Second, the topic aligned with a parallel workshop addressing methodological challenges in mHealth systematic reviews [68].

#### Workshop Analysis and Manuscript Feedback Rounds

JL-A and CMW performed a thematic analysis to summarize the workshop discussions [67] (refer to the steps in Figure 1). JL-A conducted steps 1 to 3 (familiarization with the data, initial coding from the transcription, and developing themes). CMW reviewed and refined the themes in step 4, and then JL-A and CMW defined and named the themes in step 5. Finally, all authors collaborated on step 6, organizing the final themes into a cohesive report, summarizing key insights, recommendations, and consensus points from the workshop discussions. The survey results and the workshop recommendations were integrated into the first manuscript draft, which was emailed to the workshop participants. JL-A incorporated their feedback into the final version of the manuscript.

### Ethical Considerations

We submitted the study synopsis to the Kantonale Ethikkommission (Kanton Zürich), Switzerland. After review, they stated that the study did not fall under the regulation of the Human Research Act of Switzerland (Business Administration System for Ethics Committees Req-2022-00839). The survey and meeting participants gave their consent to participate via the SoSci Survey tool. We conducted an anonymous web-based survey using the SoSci Survey platform hosted by University Hospital Zurich. Personal details such as names, email addresses, or IP addresses were not collected. In the final section of the survey, respondents were invited to participate in an optional online workshop. The responses to this invitation were stored in a separate SoSci Survey project. Participants interested in attending the workshop provided their name, email, and place of residence, among other details. To ensure privacy and confidentiality, all data were securely collected and stored at University Hospital Zurich. In addition, participants’ data were managed in strict accordance with institutional data protection policies, ensuring that their privacy was rigorously protected throughout the research process. Survey participants did not receive compensation. While workshop participants were not offered financial compensation, they were given the opportunity to be coauthors of the manuscript if they met the authorship criteria.

## Results

### Survey Results

A total of 80 authors of mHealth RCTs completed the survey. The completion rate was 40.2% (106/264, corresponding to the number of people who agreed to participate divided by the number of visitors). During testing, it consistently took <10 minutes to complete the survey. Furthermore, a similar survey conducted by our team, which had a comparable number of items but focused on challenges in systematic reviews of mHealth interventions [68], took an average of 5 (SD 2) minutes to complete, with a range of 2 to 11 minutes.

Figure 1 details the reasons for not completing the survey. The respondents’ academic backgrounds were diverse, and the most frequent were medicine (24/80, 30%), psychology (20/80, 25%), and epidemiology (12/80, 15%). Most participants (52/80, 65%) were experienced mHealth researchers who had conducted ≥2 mHealth RCTs. The survey respondents had been involved in at least 185 mHealth RCTs. Most trials (56/185, 30.3%) were conducted in North America and Europe. Few RCTs were conducted in Africa (7/185, 3.8%) and South or Central America (4/185, 2.2%; Table 1).

**Table 1:** Characteristics of the survey respondents (N=80).

Characteristic			Participants
**Academic background^a^, n (%)**			
	Medicine, nursing, or public health	32 (40)	
	Psychology or social sciences	25 (31.3)	
	Epidemiology	12 (15)	
	Physiotherapy or sports science	7 (8.8)	
	Biology or neuroscience	6 (7.5)	
	Nutrition	4 (5)	
	Engineering or computer science	5 (6.3)	
	Other backgrounds^b^	21 (26.3)	
**Clinical research experience in addition to mHealth^c^ RCTs^d^, n (%)**			
	Evaluation of nonpharmacological interventions	45 (56.3)	
	Evaluation of pharmacological interventions	0 (0)	
	Evaluation of nonpharmacological and pharmacological interventions	12 (15)	
	Other areas (eg, basic research, surveys, and prognosis research)	23 (28.8)	
**Experience in mHealth RCTs, n (%)**			
	Experienced researchers (≥2 mHealth RCTs)	52 (65)	
	Less experienced researchers (1 mHealth RCT)	28 (35)	
	Researchers involved in at least one fully remote mHealth RCT^e^	49 (61.3)	
**Number of mHealth RCTs per respondent**			
	Mean (SD)	2.3 (1.4)	
	Median (IQR)	2.3 (1-3)	
	≥5, n (%)	12 (15)	
	4, n (%)	4 (5)	
	3, n (%)	9 (11.3)	
	2, n (%)	27 (33.8)	
	1, n (%)	28 (35)	
**Location of the survey participants’ mHealth RCTs^f^ (n=185), n (%)**			
	United States	16 (8.6)	
	The Netherlands	9 (4.9)	
	Australia	8 (4.3)	
	United Kingdom and Canada (each)	7 (3.8)	
	Spain	6 (3.2)	
	China, India, and Uganda (each)	3 (1.6)	
	Finland, France, Iran, Kenya, Malaysia, New Zealand, and Sweden (each)	2 (1.1)	
	Argentina, Brazil, Germany, Guatemala, Hong Kong, Italy, Malawi, Norway, Peru, Portugal, Singapore, South Africa, South Korea, Switzerland, Taiwan, and Vietnam (each)	1 (0.5)	
Participants starting an mHealth RCT under the new Medical Device Regulation in Europe (in place since May 2021), n (%)			9 (11.3)

^a^24% (19/80) of the participants had more than one background.

^b^Other backgrounds (one participant each): behavior science, biostatistics, dentistry, design science, digital health, economics, lifestyle medicine, mathematics, medical informatics, molecular biology and biochemistry, and statistics.

^c^mHealth: mobile health.

^d^RCT: randomized controlled trial.

^e^RCT in which the recruitment, intervention delivery, and evaluation were conducted online using mobile devices or the internet.

^f^3 RCTs took place in more than one country.

Most respondents (74/80, 92%) identified at least one methodological aspect as more or much more challenging in mHealth RCTs compared to non-mHealth RCTs. Survey respondents rated a median of 6 (IQR 4-9) out of 21 aspects as more or much more challenging (Multimedia Appendix 5). The methodological aspects most frequently perceived as more or much more challenging in mHealth RCTs were the following: managing low adherence to the mHealth intervention (43/77, 56%), defining adherence (39/79, 49%), measuring adherence (33/78, 42%), analyzing passive data (eg, data collected from smartphone sensors; 24/58, 41%), determining which active components of the mHealth intervention are used or received by the participant (31/75, 41%), and verifying the participants’ identity during recruitment (participants can register more than once using different identities; 28/68, 41%). In contrast, the aspects described by the lowest number of respondents as challenging were analyzing patient-reported outcomes (6/75, 8%) and analyzing clustered data (7/62, 11%; Table 2). Multimedia Appendix 6 lists additional challenges reported by the respondents.

**Table 2:** Researchers perceiving each methodological aspect as more or much more challenging in mobile health (mHealth) randomized controlled trials (RCTs) compared to non-mHealth RCTs stratified according to experience in mHealth (N=80).

			Total sample, n/N (%)		Experienced researchers (n=52), n/N (%)^a^		Less experienced researchers (n=28), n/N (%)^a^		Absolute difference—experienced researchers – less experienced researchers (%)^b^
**Challenges in recruitment**									
	Achieving the required sample size	22/78 (28)		10/51 (20)		*12/27 (44)* ^c^		–*24*	
	Enrolling a representative sample of the desired trial population	27/77 (35)		15/50 (30)		*12/27 (44)*		–14	
	Verifying the participants’ identity during recruitment (participants can register more than once using different identities)	*28/68 (41)*		*21/45 (47)*		7/23 (30)		17	
	Implementing the informed consent procedure	10/78 (13)		6/51 (12)		4/27 (15)		–3	
**Challenges related to randomization**									
	Concealing the randomization sequence during allocation	17/76 (22)		10/49 (20)		7/27 (26)		–6	
	Including the randomization process on the app	16/55 (29)		12/37 (32)		4/18 (22)		10	
**Challenges related to intervention integrity**									
	Defining adherence to the mHealth intervention	*39/79 (49)*		*25/52 (48)*		*14/27 (52)*		–4	
	Measuring adherence to the mHealth intervention	*33/78 (42)*		*22/52 (42)*		*11/26 (42)*		0	
	Determining the active mHealth intervention components used or received by the participants	*31/75 (41)*		20/51 (39)		*11/24 (46)*		–7	
	Managing low mHealth intervention adherence rates	*43/77 (56)*		*30/52 (58)*		*13/25 (52)*		6	
**Challenges related to data quality**									
	A high proportion of participants lost to follow-up	30/78 (38)		20/52 (38)		10/26 (38)		0	
	Differential follow-up rates between intervention and comparator groups	17/70 (24)		10/47 (21)		7/23 (30)		–9	
	Suitable outcome measurement instruments for mHealth trials	20/78 (26)		17/52 (33)		3/26 (12)		*21*	
	Large amounts of missing data	31/79 (39)		18/52 (35)		*13/27 (48)*		–13	
	Verifying the validity of the data	22/76 (29)		14/51 (27)		8/25 (32)		–5	
**Challenges in data analysis**									
	Analyzing clustered data	7/62 (11)		4/42 (10)		3/20 (15)		–5	
	Analyzing large amounts of data	15/72 (21)		10/50 (20)		5/22 (23)		–3	
	Analyzing patient-reported outcomes (ie, self-reports)	6/75 (8)		4/52 (8)		2/23 (9)		–1	
	Analyzing passive data (eg, data collected from smartphone sensors)	*24/58 (41)*		*18/41 (44)*		6/17 (35)		9	
	Analyzing data using repeated measurements across time	12/75 (16)		4/51 (8)		8/24 (33)		–*25*	
	Dealing with large amounts of missing data	21/72 (29)		12/50 (24)		*9/22 (41)*		–17	

^a^Experienced mHealth researcher: involved in at least 2 mHealth RCTs. Less experienced mHealth researcher: authored 1 mHealth RCT.

^b^Absolute difference: experienced mHealth researchers (percentage) – less experienced mHealth researchers (percentage).

^c^Italics represent (1) challenges defined by at least 40% of the participants as more or much more challenging or (2) challenges with an absolute difference between experienced and less experienced mHealth researchers of >20 percentage points. Participants who responded “I don't know” or did not respond to a particular item were excluded from the analysis for that item.

Experienced and less experienced researchers’ perceptions did not differ markedly. However, a higher number of less experienced researchers considered the following aspects as more or much more challenging: achieving the required sample size (12/27, 44% vs 10/51, 20%) and analyzing data using repeated measurements across time (8/24, 33% vs 4/51, 8%). On the other hand, more experienced researchers considered the availability of suitable outcome measurement instruments for mHealth trials as more or much more challenging (17/52, 33% vs 3/26, 12%).

### Online Workshop

In total, 11 mHealth researchers attended the online workshop (2 hours; February 1, 2023; Table 3). Figure 2 outlines the topics that guided the discussion during the workshop.

**Table 3:** Characteristics of the workshop participants (N=11).

Characteristic				Participants, n (%)
Female participants				7 (64)
**Geographical area of residence**				
	**Europe**		4 (36)	
		Switzerland	2 (18)	
		Spain	1 (9)	
		United Kingdom	1 (9)	
	**North America**		2 (18)	
		Canada	1 (9)	
		United States	1 (9)	
	**Africa**		2 (18)	
		Kenya	1 (9)	
		Uganda	1 (9)	
	**Asia**		3 (27)	
		Iran	2 (18)	
		India	1 (9)	
**Number of mHealth^a^ RCTs^b^ per participant**				
	8		1 (9)	
	6		1 (9)	
	3		4 (36)	
	2		2 (18)	
	1		3 (27)	

^a^mHealth: mobile health.

^b^RCT: randomized controlled trial.

### Recommendations for Addressing Methodological Challenges Specific to mHealth RCTs

The following recommendations were agreed upon during the workshop and refined via email afterward following a qualitative process.

#### How to Measure Adherence to the mHealth Interventions?

Adherence to the intervention is the degree to which the study participants followed the intervention as intended, that is, whether the participants performed the planned intervention and avoided proscribed procedures. Measuring and reporting adherence in RCTs is essential because a low adherence can affect the validity and generalizability of the trial results [69].

##### Recommendation 1: Researchers Should Use a Variety of Methods to Assess Adherence in mHealth Trials

Several methods can measure adherence in mHealth trials, but no gold standard exists, and each method has limitations [70]. Thus, adherence in mHealth trials should be estimated from a triangulation of sources, such as the following:

- Self-report can be used for measuring adherence in mHealth trials, but this method can provide inaccurate data [71].
- Information from interviews, focus groups, or surveys serves to assess the app’s acceptability, check whether the participants adhered to the planned intervention, or test whether the participants acquired the minimum knowledge required for the mHealth intervention to have an effect.
- Direct observation is an accurate way to measure adherence, but it is also more expensive and time-consuming. This method is typically only used in small-scale studies and may not apply to mHealth RCTs.
- Aggregated and anonymous use data collected by the app (eg, tracking and dashboard mapping) are another option. Examples are the percentage of participants who opened the app or the average time spent by the participants on the app.
- Biomarkers are measurable indicators of a biological process or condition [72]. Some biomarkers can measure adherence to mHealth interventions. For example, a study used a wireless glucometer to measure adherence to diabetes self-management among patients [73]. However, biomarkers are not always available or accurate.

##### Recommendation 2: Future Research Should Inform Methodological Guidance to Link Adherence Information Collected Through Different Methods

Methodological guidance is needed to link the adherence information collected through the interaction between researchers and participants (eg, via interviews) with aggregated and anonymous adherence data routinely collected by the app (not available at the individual level due to privacy issues). Researchers should try to explain discordant adherence findings depending on the source. For example, self-declaration by the user of the time spent on the app may under- or overestimate the time registered by the app.

##### Recommendation 3: Adherence Measurement in mHealth RCTs Should Consider That Interventions Initially Delivered via the App May Not Require App Engagement at a Later Stage

Some interventions (eg, relaxation techniques or exercise) may be initially delivered via the app used in the evaluation. However, patients may learn to apply the technique alone without the app. Thus, the app is no longer used (low adherence), but the main component of the intervention (eg, relaxation techniques or exercise) is applied. Methodological guidance to measure adherence in these situations is needed.

##### Recommendation 4: The Protocol and Consent Form of mHealth RCTs Should Detail How Adherence Data Will Be Collected, Managed, and Analyzed

Trial protocols should plan data-sharing activities transparently. If adherence data will be collected via the app, the purpose of the data collection and the analysis strategy should be detailed at the protocol development stage and on the trial consent form. Use data may contain sensitive personal information. Sharing and analyzing such data without proper consent and protection may violate the participants’ privacy rights and expose them to potential risks.

##### Recommendation 5: The Trial Team Should Clarify With the App Developer Whether Data Privacy and Security Protection Issues Allow for Sharing Participants’ Use Data Between the App Developer and the Research Team

Data privacy and security protection issues may vary depending on the country, region, or institution where the trial is conducted. Different regulations and standards may apply to collecting, storing, transferring, and analyzing participants’ use data. Therefore, the trial team and the app developer should comply with the relevant laws and policies. Moreover, they should agree on the methods, formats, and frequency of data sharing, as well as the quality control and verification procedures.

##### Recommendation 6: The Measurement of mHealth Intervention Adherence Should Consider the Internet Access Requirements and Interoperability of the mHealth App

Access to the internet and interoperability should be carefully considered when measuring adherence to mHealth interventions. Researchers should differentiate between low adherence due to individual factors (eg, low app use by the participants because they did not like the app) or system factors (low app use due to connectivity difficulties).

Access to the internet is critical for mHealth intervention integrity as mHealth apps often require an internet connection to work. If users do not have good internet access, the functionality of the mHealth intervention can be compromised. In this line, poor internet access can reduce mHealth intervention integrity by worsening the app’s functioning and the participants’ adherence to the intervention.

Interoperability of the app with other software is important as this factor is related to the app’s technical functioning, user satisfaction, and adherence. For example, the app may need to work with other apps, such as calendars, reminders, and sensors, to provide optimal user experience and support.

##### Recommendation 7: Adherence Assessment in mHealth Trials Should Not Apply the Same Approaches Used in Drug Efficacy Trials

Different approaches are needed to measure adherence to mHealth interventions, and guidance is needed to tailor the measurement of adherence in mHealth trials. In this line, measures from behavior change research might be helpful in mHealth RCTs. There are critical differences concerning the assessment of adherence in RCTs of mHealth and drug interventions.

First, adherence to mHealth interventions has more dimensions than adherence to drugs. It involves the frequency and duration of app use, the quality and intensity of engagement, compliance with behavioral recommendations, and achievement of personal goals, among other aspects [74,75]. This makes it more challenging to measure adherence to mHealth interventions.

Second, adherence to mHealth interventions can be affected by the design and functionality of the app, the feedback and support provided, and the integration with other health services. Moreover, mHealth intervention adherence can vary over time and across contexts depending on the intervention duration, intensity, and frequency [76-79].

Third, the dose or course of an mHealth intervention can be adjusted according to the specific characteristics and needs of the participants. Consequently, adherence to mHealth interventions may affect the trial outcomes differently than drug adherence. For example, some participants may benefit from a short mHealth intervention, whereas others may need a longer course to achieve the desired effects [79,80].

Fourth, drug efficacy trials are typically conducted in controlled contexts, such as hospitals or specific clinical settings. This allows researchers to tightly control the environment and ensure that the intervention is delivered as intended. On the other hand, mHealth interventions are usually evaluated in real-world contexts (effectiveness), where many factors can affect adherence, such as access to the internet and technical problems. Thus, mHealth RCTs should not apply the same methods used in drug efficacy trials to assess adherence.

#### Defining Adequate Adherence

##### Recommendation 8: Predefine the mHealth Intervention Components Needed to Generate the Expected Intervention Effect and Measure Adherence Based on Those Components

mHealth interventions are multifaceted, making adherence measurement challenging. Specifying the critical mHealth intervention components (*must* items) and desirable components (*should* items) needed to generate the expected effect will help define what is adequate adherence.

There is no definitive answer to which mHealth intervention components are needed to generate the expected effects. Some factors that may contribute to the efficacy or effectiveness of mHealth interventions are the following: (1) the intervention intensity (frequency and delivery of the intervention over time); (2) its patient-centered approach (ie, tailoring the intervention content and interaction to the specific needs, preferences, and characteristics of the patients); and (3) their multifactorial approach, that is, the combination of the mHealth intervention with existing health systems and services [78,81].

If researchers are involved in designing the app or the mHealth intervention (rather than only its evaluation), they might consider conducting a scoping review to inform the logic model that outlines the components of the mHealth intervention. The selection of components should be underpinned by either existing evidence or an explanation for their choice. A logic model should also define whether the delivery sequence of the intervention components is a critical factor that can affect the outcome. If this is the case, the component sequence should also be considered to judge mHealth intervention integrity.

There is a need for a better description by app developers and researchers of each component of an mHealth intervention. Developing a common taxonomy for mHealth interventions and their components, including the behavioral techniques used, would help describe mHealth interventions in a standardized manner.

##### Recommendation 9: Future Research Should Guide How to Define Adequate Adherence in mHealth Interventions

There is no definitive answer to how to define adequate adherence to an mHealth intervention as it depends on the mHealth intervention itself, the outcome evaluated, the research aim (efficacy or effectiveness), and the measures of adherence used. The definition of adequate adherence should be based on the minimum intervention intensity or dose needed to achieve a clinically relevant effect. This information should be underpinned by existing evidence or a theoretical basis and reflect adherence to all the critical intervention components to achieve the desired effect.

The current understanding of factors that can either enhance or hinder adherence to mHealth apps remains limited, largely due to the fact that most of the underlying studies are pilot studies with short durations [70]. Future research on mHealth app adherence should clearly outline the app’s intended use; report objective data on actual use relative to the intended use; and, ideally, inform the long-term use and retention of the mHealth intervention [70].

#### Dealing With Low Adherence Rates to the mHealth Intervention

##### Overview

Adherence in mHealth intervention studies is usually low. A systematic review including 99 studies of health apps for preventing or managing noncommunicable diseases indicated that the mean adherence across all interventions was 56% (range 3%-96%) [70]. Medication adherence in drug trials seems to be higher, although this aspect is frequently not reported. For example, a systematic review on medication adherence in RCTs of patients undergoing dialysis concluded that the mean medication adherence was 81% and 84% in the intervention and control arms, respectively [82].

Addressing low adherence rates in trials is essential because low adherence can reduce statistical power and introduce bias, particularly by making a potentially efficacious intervention appear less effective. Consequently, low adherence in mHealth RCTs can lead to misleading recommendations for patients, providers, and decision makers [83].

##### Recommendation 10: Consider Using Strategies to Increase Adherence in RCTs of mHealth Interventions

Researchers may consider using different strategies to increase adherence in mHealth RCTs (with the caveats addressed in recommendation 11). Possible strategies are the following [84-87]. The first is to provide real-time or regular feedback to the participants based on their performance, such as congratulating them for achieving their goals, encouraging them to improve their adherence, or suggesting tips or strategies to overcome barriers. The second is to send reminders to the participants via SMS text messages, voice calls, or push notifications to help them remember to use the mHealth app. The third is to apply behavior change techniques to enhance the participants’ motivation and self-efficacy to adhere to the mHealth intervention. The fourth is to tailor the mHealth intervention to the specific needs and preferences of the participants, such as allowing them to choose the frequency, timing, content, and format of the messages or feedback or personalizing the intervention based on their demographic, clinical, or behavioral characteristics. The fifth is to integrate the mHealth intervention with the existing health systems and services, such as ensuring the coordination of the mHealth app with health care providers or involving health care providers in delivering or supporting the intervention.

##### Recommendation 11: Define the Role of the Strategy to Increase Adherence to the mHealth Intervention

The trial protocol should specify the function of the strategy to enhance adherence to the mHealth intervention. Whether this strategy is an integral part of the mHealth intervention or a measure implemented merely in the RCT to boost adherence needs to be determined.

Researchers should be aware that incorporating strategies to improve adherence in RCTs can bias the study results, especially in studies with active comparators that did not use these strategies. Therefore, if one group receives additional strategies to improve adherence but the other group does not, this could introduce a performance bias [88]. Furthermore, if the strategy is not going to be a component of the mHealth intervention in real-world settings, the adherence observed in the RCT may not accurately represent adherence in actual settings. Researchers should carefully consider the ethics of using incentives to boost adherence in mHealth RCTs. While incentives are not inherently unethical, ethical concerns primarily emerge from their large-scale implementation in real-world scenarios. This is especially true if the incentives are expensive or challenging to sustain, raising issues regarding their long-term viability [4,5].

##### Recommendation 12: Involve Experts in Clinical Trial Design and Analysis to Handle High Amounts of Missing Participant Data and Low Adherence to the mHealth Intervention

Missing participant data, that is, missing outcome data for participants in a trial, is a common problem in RCTs. Missing participant data can occur when participants drop out of the study, fail to complete specific assessments, or are lost to follow-up [89]. Missing data problems are likely exacerbated in mHealth RCTs and affect their validity [90]. For example, early dropouts (recruited participants who did not start using the intervention) are probably a common and essential issue in mHealth RCTs [91]. Moreover, as stated previously, low adherence rates are frequent in mHealth RCTs [70].

mHealth RCTs should involve statisticians and experts in trial design and analysis to implement appropriate strategies to handle and analyze high amounts of missing participant data and low adherence. There is no agreed upon guidance, but the implemented approach should follow these general principles: (1) anticipate high amounts of missing participant data in the sample size calculation, (2) clearly report missing participant data (numbers and reasons per study arm), (3) conduct the primary analysis under the most plausible assumption as to why the data are missing, and (4) conduct sensitivity analyses considering alternative plausible assumptions to assess the robustness of the conclusions [89,92]. The importance of tracking adherence metrics and understanding the factors that lead to participant dropout should be quantified, analyzed, discussed, and reported. This also involves examining the attributes of the subset of participants who remain in the trial and use the mHealth intervention [91].

#### Addressing mHealth Intervention Components

##### Overview

The turnover in mHealth app development is fast. The rate at which mHealth apps are created, updated, or discontinued by developers is high. Consequently, obtaining relevant data to support future research and decision-making can be complex because critical information about the app (or the version evaluated in a trial) may no longer be available. However, the critical set of information that should be shared is still to be agreed upon.

##### Recommendation 13: Report the App’s Technical Specifications and Cost and the Regulations Fulfilled During Its Development and Evaluation

It is essential to report the technical specifications of the app evaluated in the trial, such as version, launch date, compatibility with different devices and operating systems, storage, and security features. The cost of the app is also important, including any subscription or in-app purchase fees. All this information helps assess the accessibility of the app, particularly for populations with limited financial resources.

mHealth app development is subject to regulations and standards from different authorities and organizations, such as compliance with data collection and storage practices and data protection regulations. Knowing these regulations is vital for future researchers and app users as they impose specific requirements and limitations that may affect the apps’ use.

##### Recommendation 14: App Developers and Researchers Should Report Users’ Feedback Regarding the mHealth Intervention

App developers and researchers should report the findings of user-centered methods used to design or evaluate the mHealth intervention, for example, focus groups on the app’s usability. Critical information could include the users’ views on app functionality, usability, privacy, security, and quality [93]. In addition, the users’ literacy with the mHealth intervention during the trial should be reported.

User feedback can provide valuable insights such as the following to understand the role of each mHealth intervention component and explain adherence: (1) the processes, mechanisms, and outcomes of mHealth interventions, which can help improve their design, implementation, and evaluation; (2) the strengths and weaknesses of the mHealth intervention, such as its usability, functionality, acceptability, and effectiveness, from the perspective of the end users; and (3) barriers and facilitators that influence the adoption, engagement, and retention of mHealth interventions. User feedback can contribute to the knowledge translation of mHealth interventions by sharing the experiences, opinions, and preferences of the target population with other researchers and stakeholders [93-97].

##### Recommendation 15: Consider Incorporating Scaling Lessons Learned in the Technology Field Into the mHealth Intervention Development Process and Evaluation

Traditional health intervention evaluation often relies on lengthy, static RCTs. However, this method fails to capture the dynamic nature of mHealth app development. In contrast, technology companies such as Amazon use agile online randomized experiments on a large scale, running hundreds of simultaneous controlled experiments on millions of users. This approach is used to launch new products, assess their value, enhance customer experience, and implement code modifications. A/B testing is one such method. It is a quick experimentation process that efficiently evaluates multiple design options using live environments and real users. Essentially, A/B testing compares 2 versions of a variable to determine which one performs better [98-100].

Developers and researchers should consider using A/B testing methods to evaluate the impact of different features of the mHealth intervention. A/B testing can help identify app attributes that promote adherence and positive outcomes, minimize unintended consequences, or predict poor user experience. This approach can be applied to both major and minor design choices, such as the placement of icons on the app. Therefore, mHealth app developers and researchers should consider the involvement of experts who can analyze large amounts of data per user, swiftly implement improved versions, assess the relative impact of the modifications, and then modify the product for the next round [100]. However, A/B testing methods can be expensive and require that the individuals conducting the trial are also able to change targeted aspects of the software.

##### Recommendation 16: The Impact of App Updates Should Be Carefully Considered in RCTs of mHealth Interventions

App updates often include changes to the app’s functionality and user interface and bug fixes. If some users have updated versions whereas others do not, it could introduce variability in data collection and user experience, potentially impacting the overall findings. For these reasons, mHealth RCTs must handle app updates carefully to ensure valid and reliable results. This information will help evaluators, users, and prescribers judge whether the mHealth intervention has evolved into a different intervention.

It is important to define the key aspects (a core set of items) that should be described regarding app updates. Some of these items for mHealth RCTs may be the following: (1) the version that was evaluated and whether newer app versions were implemented during the trial; (2) implemented app updates—key mHealth intervention components that were modified along the trial, reasons, and periodicity; and (3) app changes that were considered major changes (distinguishing between technical updates and content updates).

The RCT protocol should plan how to manage the app updates, and the ethics submission should also describe relevant aspects, such as the app changes that will be communicated to the ethics committee or the ethical implications of the use of apps that are not updated. It is also important that researchers and app developers collaborate and clarify the potential implications of updating the app for the validity and reliability of the study results.

##### Recommendation 17: The RCT Protocol Should Carefully Choose and Define the Control Intervention

Control conditions in mHealth trials are crucial for establishing the efficacy of the intervention. Each type of comparator has its strengths and limitations, and its choice should be carefully based on the research question, ethical considerations, and the nature of the mHealth intervention [101,102]. The following are some types of control conditions that can be used in mHealth trials.

- Sham control—a control group with a placebo or inactive treatment. This type of control can be used in mHealth trials in which the control group might receive a sham app with no active ingredients.
- Active control—a control group that is given an existing, proven intervention. This type of control is often used when it would be unethical to give a placebo, such as when an effective treatment already exists.
- A/B testing—the control group is another version of a variable to determine which one performs better (recommendation 15).
- Waitlist control—a control group that receives the intervention after the active group has already received it. This type of control is often used in psychological and behavioral interventions.
- Attention control—a control group that receives an intervention that is intended to control for the amount of attention received by the active group.
- Treatment as usual—a control group that continues to receive the standard care or treatment as usual while the experimental group receives the new intervention.

## Discussion

### Principal Findings

We contacted 1535 authors of mHealth intervention RCTs, of whom 80 (5.21%) completed the survey. Our research identified specific methodological challenges in RCTs of mHealth interventions. The aspects most frequently reported as challenging were those related to mHealth intervention integrity, in particular managing low adherence to the mHealth intervention, defining adherence, measuring adherence, and determining which mHealth intervention components are used or received by the participants. Other challenges were also frequent, such as analyzing passive data and verifying the participants’ identity during recruitment to the RCT. The workshop concentrated on the integrity of mHealth interventions. Following the workshop and subsequent email discussions, we established 17 consensus-based recommendations to address selected methodological challenges related to mHealth intervention integrity.

### Strengths and Limitations

Our study has several strengths. First, we likely identified the main methodological hurdles specific to mHealth RCTs. The process began with nonsystematic literature searches that informed the potential challenges listed in the survey. Interestingly, survey respondents identified only 1 methodological challenge not included in our survey: finding a suitable control condition, such as creating a placebo app. Second, the survey and workshop participants had diverse experiences, having been involved in 1 to >5 mHealth RCTs each. This range of expertise allowed our study to encapsulate the challenges encountered by mHealth researchers with varying experience levels. Furthermore, the workshop participants were involved in mHealth RCTs conducted in high- and low-resource settings, such as the United States, Argentina, Uganda, and Iran. This diversity probably helped capture the challenges that researchers face in different contexts. Third, a key strength of our project was adherence to reporting guidelines for online surveys and consensus documents. This alignment with best practices enhanced the credibility and reproducibility of our work (Multimedia Appendices 7 and 8).

This study has several limitations. First, the low response rate may have affected the representativeness of the results as participants may have had a specific interest or experience in mHealth RCTs. Contributing factors include inoperative emails, out-of-office replies, the specificity of the topic, and survey fatigue. Online surveys generally suffer from lower response rates compared to other methods [103,104]. In our survey, despite sending reminders and attempting to reach a diverse pool of researchers, this limitation persisted. Future studies could use additional strategies such as incentives or targeted recruitment to improve participation rates.

Second, only 15% (12/80) of the respondents had experience with both mHealth and non-mHealth interventions, limiting the survey’s ability to capture the challenges unique to mHealth RCTs. Including more researchers with experience in both areas could provide a more comprehensive understanding of these challenges in future studies.

Third, the study did not account for differences between various types of mHealth interventions. These interventions vary significantly, ranging from simple SMS text messaging to complex, multicomponent apps, each presenting distinct challenges. This variability may have influenced the findings. Future research should categorize mHealth interventions to provide more targeted recommendations for addressing specific challenges.

Fourth, the complexity of measuring adherence in mHealth RCTs was not fully explored. Adherence is difficult to measure due to factors such as user engagement, app use, and internet access. While adherence was identified as a key challenge, more research is needed to develop standardized methods for its measurement. Future studies should also examine how adherence differs across various types of mHealth interventions and how it impacts study outcomes.

### Comparison With Prior Work

To our knowledge, our survey is the first to ask mHealth researchers about methodological challenges specific to mHealth RCTs. Our findings align with those of previous research on the challenges in mHealth intervention research. A review of RCTs evaluating the effects of mHealth interventions in chronic disease management identified the following challenges as the most important ones: designing high-quality studies, developing robust interventions in combination with health professional input, and identifying tools and methods to improve patient adherence [105]. Another systematic review pinpointed methodological challenges in RCTs of smartphone-based interventions in psychiatry. These included the need for flexible trial designs that can quickly adapt to the fast-evolving nature of mHealth interventions, privacy and security concerns, the lack of standardized adherence metrics, and the difficulties in choosing primary outcomes and handling missing data [24].

Our research advances mHealth methodology by addressing specific challenges not comprehensively covered in other studies [24,25,105]. First, we examined intervention integrity, highlighting the intricacies of defining and measuring adherence and pointing to the need to define the essential mHealth intervention components. Second, we spotlighted the challenge of analyzing passive smartphone sensor data, a significant digital health issue overlooked in previous research focused on traditional data analysis challenges. Third, our work underscores the unmet need for effective digital identity verification in studies relying on online recruitment, a crucial aspect often overlooked in previous research in favor of recruitment strategies and sample size considerations.

Our study sets itself apart from previous research by calling for a clear definition of mHealth intervention adherence, emphasizing the need to compare between the actual and intended use of the intervention against predefined thresholds. Previous research has broadly addressed mHealth adherence challenges, noting that poor reporting standards in mHealth RCTs hinder adherence assessment [24,70,106]. In addition, our study distinguishes itself from previous research by advocating for a transparent framework that requires researchers to predefine the intended use of the mHealth intervention and choose clear thresholds for adherence evaluation. However, we recognize our limitation in not offering specific guidelines for measuring adherence, for example, by proposing adherence thresholds. Nevertheless, advocating for a transparent and operative framework to measure mHealth intervention adherence is a first important step.

Most of the methodological challenges that this study highlighted are associated with mHealth intervention integrity. There is no gold standard for measuring adherence in mHealth trials [70,107]. This construct is challenging to define and measure in mHealth trials for several reasons. First, mHealth trials have different factors influencing adherence compared to drug interventions, such as more diverse participant characteristics, preferences, and context, such as internet access [19]. The key difference likely stems from drug interventions occurring in controlled settings versus mHealth interventions occurring in real-world settings, which are less controlled and more variable [8,9]. Second, adherence can be measured at different trial phases, such as at intervention initiation (when the participants begin the intervention), during the implementation period, or at discontinuation (the permanent cessation of the randomized intervention) [108]. Third, the data collection sources and methods to measure adherence are diverse, such as self-reporting, interviews, or electronic tracking via mobile devices [109]. In this line, adherence definitions and thresholds can vary, such as the number of uses of the app, the time spent using the app, or the proportion of participants who achieved a certain level of adherence. Fourth, mHealth trials have different factors influencing adherence compared to drug interventions, such as more diverse participant characteristics, preferences, and context (eg, access to the internet network connectivity) [19].

Consequently, mHealth intervention integrity is seldom discussed in RCTs, likely due to the absence of universally accepted definitions and measurements [69]. Furthermore, existing reporting guidelines do not advise on how to report the integrity of mHealth interventions [25,110-114], and it remains uncertain how mHealth intervention integrity information should be managed by researchers and decision makers. Nevertheless, mHealth RCTs should strive to assess the integrity of interventions for each study arm and incorporate these data into their analyses, possibly using standardized data extraction forms. In this regard, a quantitative framework for evaluating the integrity of mHealth interventions could promote uniform assessment in RCTs. However, this is a complex task as there is no consensus on the model variables and their respective weights [69].

### Conclusions

RCTs of mHealth interventions have specific methodological challenges compared to those of non-mHealth interventions, particularly those related to intervention integrity. Following our recommendations for addressing these challenges can lead to more reliable assessments of the effects of mHealth interventions on health outcomes.

## Appendix

Multimedia Appendix 1 References of documents on research methods consulted for methodological challenges.

Multimedia Appendix 2 Invitation email and web-based survey.

Multimedia Appendix 3 Identification of authors in Web of Science.

Multimedia Appendix 4 Workshop slides.

Multimedia Appendix 5 Survey dataset.

Multimedia Appendix 6 Additional challenges.

Multimedia Appendix 7 Checklist for Reporting Results of Internet E-Surveys.

Multimedia Appendix 8 Checklist for Accurate Consensus Reporting Document guidelines.

## Abbreviations

CONSORT-EHEALTH: Consolidated Standards of Reporting Trials of Electronic and Mobile Health Applications and Online Telehealth
EQUATOR: Enhancing the Quality and Transparency of Health Research
mHealth: mobile health
RCT: randomized controlled trial
