# Supplementary material for: Methodological Challenges in Randomized Controlled Trials of mHealth Interventions: Cross-Sectional Survey Study and Consensus-Based Recommendations
Source: J Med Internet Res. 2024 Dec 19;26:e53187. doi: 10.2196/53187 (PMC11695959; doi:10.2196/53187)
Supplement: Multimedia Appendix 2 [file jmir_v26i1e53187_app2.pdf]

Appendix 2: Invitation Email and Web-based Survey

From: IKI Survey
To: IKI Survey
Subject: Participate in the development of mHealth trial methods recommendations
Date: 23 September 2022 14:28:19

Dear researcher

We identified you through a search in Web of Science for mHealth intervention trials.

We invite you to participate in our survey to identify methodological challenges in randomized trials evaluating the effects of mHealth interventions. The survey does not cover mHealth in other clinical scenarios, such as health monitoring or diagnosis.

Click here to participate: https://befragung.usz.ch/mHMC/

We will use the survey results to inform a consensus workshop. The aim of this workshop will be to develop recommendations to address methodological challenges in randomized trials of mHealth interventions. At the end of this survey, you can indicate whether you are interested in participating in this consensus workshop.

This survey is part of the NIH-funded project: Cochrane Complementary Medicine Field: Resource for Research (2R24AT001293).

The survey is **anonymous** and will take you a **maximum of ten minutes**. You can fill in part of the survey, save it with the button "Pause the interview", and complete it later.

If you have any questions, don’t hesitate to get in touch with us: survey@usz.ch

Claudia Witt, MD, MBA, **L Susan Wieland**, MPH, PhD & **Jesús López-Alcalde**, MPH

Cochrane Complementary Medicine, University of Maryland School of Medicine, USA & University of Zurich, Switzerland

Click here to participate: https://befragung.usz.ch/mHMC/

Online survey: Methodological challenges in randomized trials of mHealth Interventions

Dear researcher

We identified you through a search in Web of Science for mHealth intervention trials.

We invite you to participate in our survey to identify methodological challenges in randomized trials evaluating the effects of mHealth interventions. The survey does not cover mHealth in other clinical scenarios, such as health monitoring or diagnosis.

We will use the survey results to inform a consensus workshop. The aim of this workshop will be to develop recommendations to address methodological challenges in randomized trials of mHealth interventions. At the end of this survey, you can indicate whether you are interested in participating in this consensus workshop.

This survey is part of the NIH-funded project: Cochrane Complementary Medicine Field: Resource for Research (2R24AT001293).

The survey is **anonymous** and will take you a **maximum of ten minutes**. You can fill in part of the survey, save it with the button "Pause the interview", and complete it later.

If you have any question, don't hesitate to get in touch with us: survey@usz.ch

Claudia Witt, MD, MBA, **L Susan Wieland**, MPH, PhD & **Jesús López-Alcalde**, MPH

Cochrane Complementary Medicine, University of Maryland School of Medicine, USA & University of Zurich, Switzerland

Consent to participate in the survey

You are eligible to participate in this survey if you have been involved in the methodological aspects of at least one randomized trial of a mHealth intervention. Mobile Health, also known as mHealth, is the medical and public health practice supported by mobile devices. Examples of mobile devices are smartphones or tablets.

"I confirm that I meet the requirements to participate in the survey and I agree to take part."

- ◯ Yes
- ◯ No

Methodological challenges in mHealth randomized trials

You can see below a list of methodological aspects that may be challenging in randomized trials of mHealth interventions when compared to other established research fields, such as drug evaluation or psychotherapy trials. Please remember that mHealth in other clinical scenarios, such as health monitoring or diagnosis, is not the scope of this survey.

How challenging did you find the methodological aspects in your mHealth randomized trial(s) compared to your experience or observation of non-mHealth trials?

Challenges in the recruitment

How challenging did you find recruitment in your mHealth trial(s) compared to non-mHealth trials?

	much less challenging	less challenging	similar challenges	more challenging	much more challenging	don't know
Achieving the required sample size	○	○	○	○	○	○
Enrolling participants with characteristics representative of the desired trial population	○	○	○	○	○	○
Verifying the participants' identity (participants can register more than once using different identities)	○	○	○	○	○	○
Implementing the informed consent procedure	○	○	○	○	○	○

Challenges in the randomization

How challenging did you find randomization in your mHealth trial(s) compared to non-mHealth trials?

	much less challenging	less challenging	similar challenges	more challenging	much more challenging	don't know
Concealing the randomization sequence during allocation to treatment	○	○	○	○	○	○
Including the randomization process into the app	○	○	○	○	○	○

Challenges related to the intervention integrity

How challenging did you find intervention integrity in your mHealth trial(s) compared to non-mHealth trials?

	much less challenging	less challenging	similar challenges	more challenging	much more challenging	don't know
Defining adherence to the mHealth intervention	○	○	○	○	○	○
Measuring adherence to the mHealth intervention	○	○	○	○	○	○
Determining which active components of the mHealth intervention are used/received by the participant	○	○	○	○	○	○
Low rates of adherence to the mHealth intervention	○	○	○	○	○	○

Challenges related to data quality

How challenging did you find participants' retention and outcomes measurement in your mHealth trial(s) compared to non-mHealth trials?

	much less challenging	less challenging	similar challenges	more challenging	much more challenging	don't know
High proportion of participants lost-to-follow-up	○	○	○	○	○	○
Differential follow-up rates between the intervention and comparator groups	○	○	○	○	○	○
Availability of suitable outcome measurement instruments for mHealth trials	○	○	○	○	○	○
Large amount of missing data	○	○	○	○	○	○
Verifying the validity of data	○	○	○	○	○	○

Challenges in data analysis

How challenging did you find data analyses in your mHealth trial(s) compared to non-mHealth trials?

	much less challenging	less challenging	similar challenges	more challenging	much more challenging	don't know
Analyzing clustered data	○	○	○	○	○	○
Analyzing large amounts of data	○	○	○	○	○	○
Analyzing patient-reported outcomes (i.e., self-reports)	○	○	○	○	○	○
Analyzing passive data (e.g., data collected from smartphone sensors)	○	○	○	○	○	○
Analyzing data with repeated measurements across time	○	○	○	○	○	○
Dealing with large amounts of missing data	○	○	○	○	○	○

Please, add any other methodological challenges in mHealth randomized trials or any other comment you would like to share

What is your academic background?

(You can choose multiple answers)

- ☐ Biology
- ☐ Chemistry
- ☐ Computer science
- ☐ Economics
- ☐ Engineering
- ☐ Epidemiology
- ☐ Linguistics
- ☐ Medicine
- ☐ Neuroscience
- ☐ Nursing
- ☐ Philosophy
- ☐ Physics
- ☐ Psychology
- ☐ Physiotherapy
- ☐ Social science
- ☐ Sports science
- ☐ Other
  please specify

How many randomized trials on mHealth interventions have you been involved in?

[Please choose]

Have any of your trials been fully remote? (participation, delivery of intervention and evaluation conducted virtually using mobile devices or the internet)

- ○ Yes
- ○ No

In which countries did the trial/s take place?

(You can choose multiple answers)

- ☐ Australia
- ☐ Canada
- ☐ China
- ☐ Finland
- ☐ France
- ☐ Germany
- ☐ Netherlands
- ☐ New Zealand
- ☐ Norway
- ☐ Spain
- ☐ Switzerland
- ☐ United Kingdom
- ☐ United States
- ☐ Other please specify

How would you describe your clinical research besides trials on mHealth interventions? (Please choose one option)

- ○ Mainly evaluation of non-pharmacological interventions
- ○ Mainly evaluation of pharmacological interventions
- ○ Non-pharmacological and pharmacological interventions
- ○ Mainly other research areas, such as basic research, epidemiology, surveys, prognosis research, diagnostic tests, etc.

Have you started any mHealth trial under the new Medical Device Regulation in Europe (in place since May 2021)?

- ○ Yes
- ○ No

Cochrane Complementary Medicine will organize a two- or three-hour online workshop to develop recommendations for addressing methodological challenges in randomized trials of mHealth interventions. The date is yet to be confirmed but the workshop will be held sometime between November 2022 and March 2023.

Would you be interested in participating?

- ○ Yes (by selecting this option you leave the anonymous part of the survey)
- ○ No

Thank you for your interest in our workshop.

Please fill out a contact form, which can be found at **https://befragung.usz.ch/ContactForWorkshop_mHMC/**

Note: If clicking on the link does not work, please copy the link in a new browser window and enter it in there.

Thank you for completing this survey!

If you have any questions, please contact survey@usz.ch

You can close the browser window or tab now.

Thank you for your interest in our upcoming online workshop

By completing the following questionnaire, I agree that the information provided can be used to invite me to a workshop. Its aim will be to develop recommendations to overcome methodological challenges in randomized trials of mHealth interventions.

The 2-3 hours online workshop will be organized by Cochrane Complementary Medicine between November 2022 and March 2023 (date to be confirmed).

If you know other colleagues who want to participate, please feel free to share this link:

https://befragung.usz.ch/ContactForWorkshop_mHMC/

If you have any questions, please get in touch with survey@usz.ch

Title

First name

Surname

Affiliation

Country

Email

Are there topics you are especially interested to discuss?

Thank you for completing the form!

We are looking forward to get in touch with you with more details on the upcoming online workshop. Meanwhile, if you have any questions or further topics, please do not hesitate to contact us: survey@usz.ch

Your answers were transmitted, you may close the browser window or tab now.
