# Supplementary material for: Methodological Challenges in Randomized Controlled Trials of mHealth Interventions: Cross-Sectional Survey Study and Consensus-Based Recommendations
Source: J Med Internet Res. 2024 Dec 19;26:e53187. doi: 10.2196/53187 (PMC11695959; doi:10.2196/53187)
Supplement: Multimedia Appendix 3 [file jmir_v26i1e53187_app3.docx]

## Appendix 3. Identification of authors of mHealth trials

**Aim:** To collect contact emails of authors of randomized controlled trials (RCTs) of mHealth interventions indexed in Web of Science between 1 January 2018 and 17 June 2022.

The search strategy contains terms related to RCTs and systematic reviews (SRs), as we also used its results to inform a survey of methodological challenges of mHealth SRs.

**Step 1. Search in MEDLINE (via PubMed), accessed 17 June 2022**

| #1 | "Mobile Applications"[Mesh] | | | 10,129 |
| --- | --- | --- | --- | --- |
| #2 | (mobile*[ti] OR phone*[ti] OR telephone*[ti] OR smartphone*[ti] OR cellphone*[ti] OR smartwatch*[ti] OR Software[ti] OR Portable[ti]) AND (application*[ti] OR app[ti] OR apps[ti]) | | | 7,384 |
| #3 | ("mobile health"[ti] OR mhealth[ti] OR m-health[ti] OR ehealth[ti] OR e-health[ti]) | | | 7,129 |
|  |  | | | 19,815 |
| #4 | #1 OR #2 OR #3 |  |  |  |
| #5 | #4 AND (randomized controlled trial[Publication Type] OR (randomized[Title/Abstract] AND controlled[Title/Abstract] ANDtrial[Title/Abstract])) OR systematic[sb] | | | 3,091 |

**Step 2. Download the results in PMID format**

We opened a Microsoft Word file to modify and replace the line breaks with OR.

**Step 3. Search in WoS (Science Citation Expanded), accessed 17 June 2022**

| #1 | Search records with PMIDs in WoS | 2,818 |
| --- | --- | --- |
| #2 | TI=(“Mobile Application*” OR “Cell Phone*” OR cellphone* OR smarthphone* OR “Handheld computer*” OR smartwatch* OR app OR apps OR “mobile health” OR mhealth OR m-health OR ehealth OR e-health) OR AK=(“MobileApplication*” OR “Cell Phone*” OR cellphone* OR smarthphone* OR “Handheld computer*” OR smartwatch* OR app OR apps OR “mobile health” OR mhealth OR m-health OR ehealth OR e-health) OR KP=(“Mobile Application*” OR “CellPhone*” OR cellphone* OR smarthphone* OR “Handheld computer*” OR smartwatch* OR app OR apps OR “mobilehealth” OR mhealth OR m-health OR ehealth OR e-health) | 37,171 |
| #3 | ALL=("clinical trial*" OR random* OR placebo OR trial* OR systematic* OR scoping OR metanalysis OR meta-analysis OR Clinicaltrials.gov OR NCT* OR ICTRP OR “International Clinical Trials Registry Plataform” OR EU-CTR OREU-CTIS OR “EU Clinical Trials registry” OR EudraCT OR “EEA CTA” OR UTN OR “Universal Trial Number” OR PROSPERO) | 3,871,758 |
| #4 | #2 AND #3 | 8,468 |
| #5 | #4 AND (Neurology OR Oncology or Nursing OR Pharmacology Pharmacy OR Nutrition Dietetics OR Endocrinology Metabolism OR Pediatrics OR Psychology OR Obstetrics OR Gynecology OR Rehabilitation OR Psychology Clinical OR Surgery OR Geriatrics Gerontology OR Infectious Diseases OR Substance Abuse OR Peripheral Vascular Disease OR Respiratory System OR Sport Sciences OR Orthopedics OR Urology Nephrology OR Rheumatology OR Engineering Biomedical OR Gastroenterology OR Hepatology OR Psychology Multidisciplinary OR Dermatology OR Allergy OR Dentistry Oral Surgery Medicine OR Otorhinolaryngology OR Anesthesiology OR Gerontology ORPrimary Health Care OR Critical Care Medicine OR Emergency Medicine OR Audiology Speech Language Pathology OR Hematology OR Tropical Medicine OR Integrative Complementary Medicine v Ophthalmology) (Web of Science categories) | 6,256 |
| #6 | #1 OR #5 | 7475 |

**Step 4. Identification of mHealth RCTs published between 2018 and 2022**

We identified a total of 1,735 eligible mHealth RCTs. Bibliographic details are available upon request.

**Step 5. Identification of authors of RCTs of mHealth interventions**

We could download the emails of 1,535 authors of these RCTs.
