# Supplementary material for: Methodological Challenges in Randomized Controlled Trials of mHealth Interventions: Cross-Sectional Survey Study and Consensus-Based Recommendations
Source: J Med Internet Res. 2024 Dec 19;26:e53187. doi: 10.2196/53187 (PMC11695959; doi:10.2196/53187)
Supplement: Multimedia Appendix 4 [file jmir_v26i1e53187_app4.pdf]

Cochrane Complementary Medicine

Trusted evidence.
Informed decisions.
Better health.

Online workshop: Methodological challenges in randomised controlled trials of mHealth interventions

1 February 2023

13:00 – 15:00 h CET

Claudia M. Witt
L. Susan Wieland
Jesús López-Alcalde

Cochrane Complementary Medicine
University of Maryland School of Medicine
University of Zurich and University Hospital Zurich

USZ Universitäts Spital Zürich

Universität Zürich[UZH]

University of Maryland School of Medicine

Recording of the workshop

The workshop will be recorded for summarising the results.
The recording will be only used internal purposes.

If anyone objects, please speak up.

Agenda

1 Feb 2023. 13:00 – 15:00 h CET

Cochrane Complementary Medicine

1. **Introduction of participants**
   13:00 – 13:15
2. **Project presentation**
   13:15 – 13:30
   Jesús López-Alcalde
3. **Developing guidance**
   13:30 – 14:50
   Claudia M. Witt
4. **Closing remarks**
   14:50 – 15:00
   L. Susan Wieland

USZ Universitäts Spital Zürich — Universität Zürich[UZH] — University of Maryland School of Medicine

1. Introduction of participants

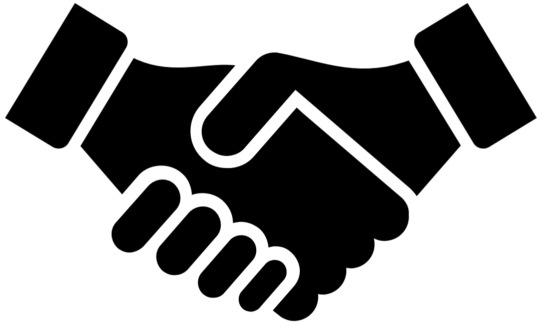

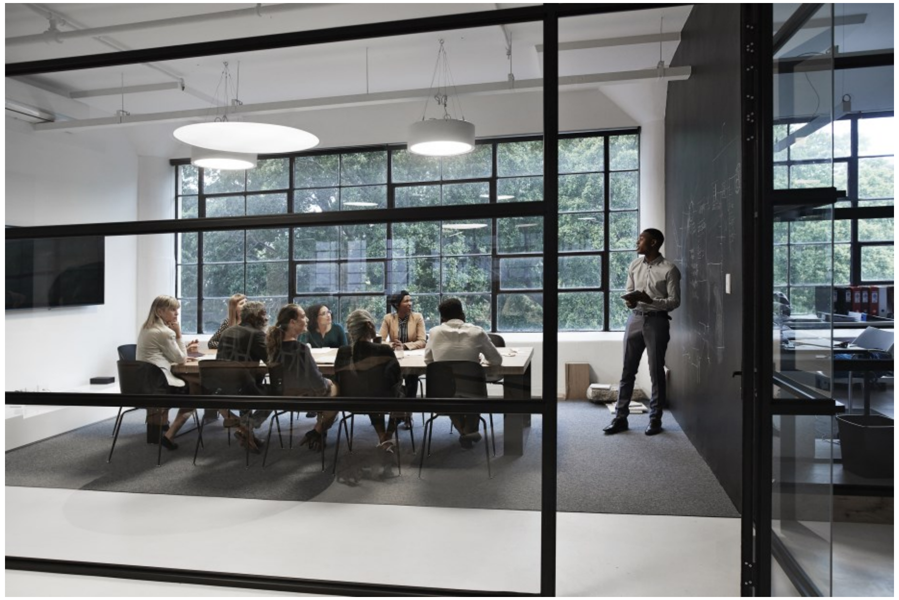

- Name
- Country and institution
- Experience in RCTs of mHealth interventions

Agenda

1 Feb 2023. 13:00 – 15:00 h CET

Cochrane Complementary Medicine

1	2	3	4
**Introduction of participants**	**Project presentation**	**Developing guidance**	**Closing remarks**
13:00 – 13:15	13:15 – 13:30	13:30 – 14:50	14:50 – 15:00
	Jesús López-Alcalde		

USZ Universitäts Spital Zürich

Universität Zürich[UZH]

UNIVERSITY of MARYLAND SCHOOL OF MEDICINE

a. Scope of the survey

01 Clinical question — **Effects of health interventions**

- Other clinical questions excluded
- Examples: health monitoring, dx or prognosis

02 Intervention — **Mobile Health (mHealth) interventions**

- Health interventions supported by mobile devices

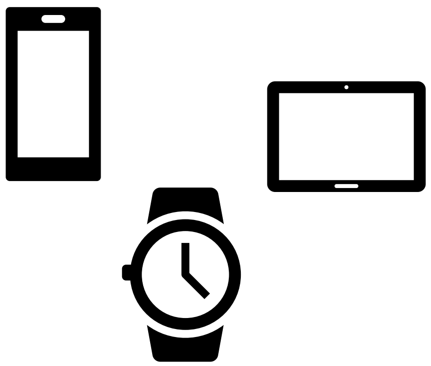

03 Design — **Randomised controlled clinical trial (RCT)**

04 Survey aim — To **identify methodological challenges** specific to RCTs evaluating the effects of mHealth interventions

b. Timeline: work done and following steps

Sept – Nov 2022

To **identify** methodological challenges in RCTs of mHealth interventions

2023

To **develop recommendations** to overcome the most relevant challenges identified

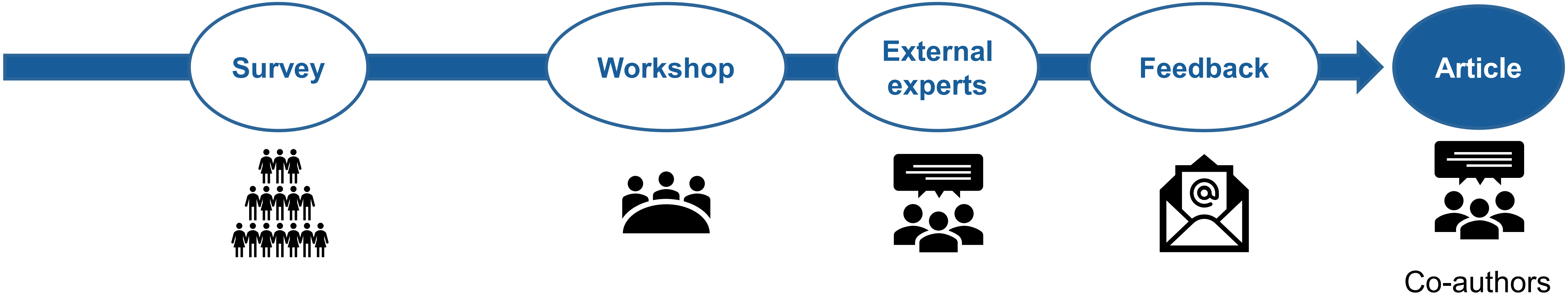

c. Methodological challenges in RCTs of mHealth interventions: survey preliminary results

01 Survey sample size:

- Total participants: 79
- Experienced trialists (authoring ≥2 RCTs): 52

02 Most challenging areas in RCTs of mHealth interventions:

Aspects related to the mHealth intervention itself

Intervention integrity

Criteria for workshop topic selection:

1. Answers of experienced reviewers
2. Topics uncovered in relevant methodological guidance*:
   - mERA reporting checklist
   - CREMAIs reporting guideline
   - CONSORT-EHEALTH
   - TIDieR-telehealth
   - MARS (Mobile app rating scale)

* Based on a non-systematic review

Concept: intervention integrity

Proposed definition

The degree to which the study intervention was delivered as intended[1-5]

Components of intervention integrity

- **Intervention delivery** ⇔ **Participant receiving the intervention**

Intervention delivery:
- Device
- App
- Internet access
- Health provider

Participant receiving the intervention:
- Adherence
- Withdrawals

USZ Universitäts Spital Zürich

Universität Zürich[UZH]

[1] Perepletchikova F. Clin Psychol (New York). 2011;18(2):148-153.
[2] Perepletchikova F, et al. J Consult Clin Psychol. 2009;77(2):212-218.
[3] Perepletchikova F, et al. J Consult Clin Psychol. 2007;75(6):829-841.
[4] Yeaton WH, et al. J Consult Clin Psychol. 1981;49(2):156-167.
[5] Lopez-Alcalde J, et al. J Clin Epidemiol. 2022;151:65-74.

% Experienced trialists considering each aspect as "More challenging" or "Much more challenging" in mHealth RCTs

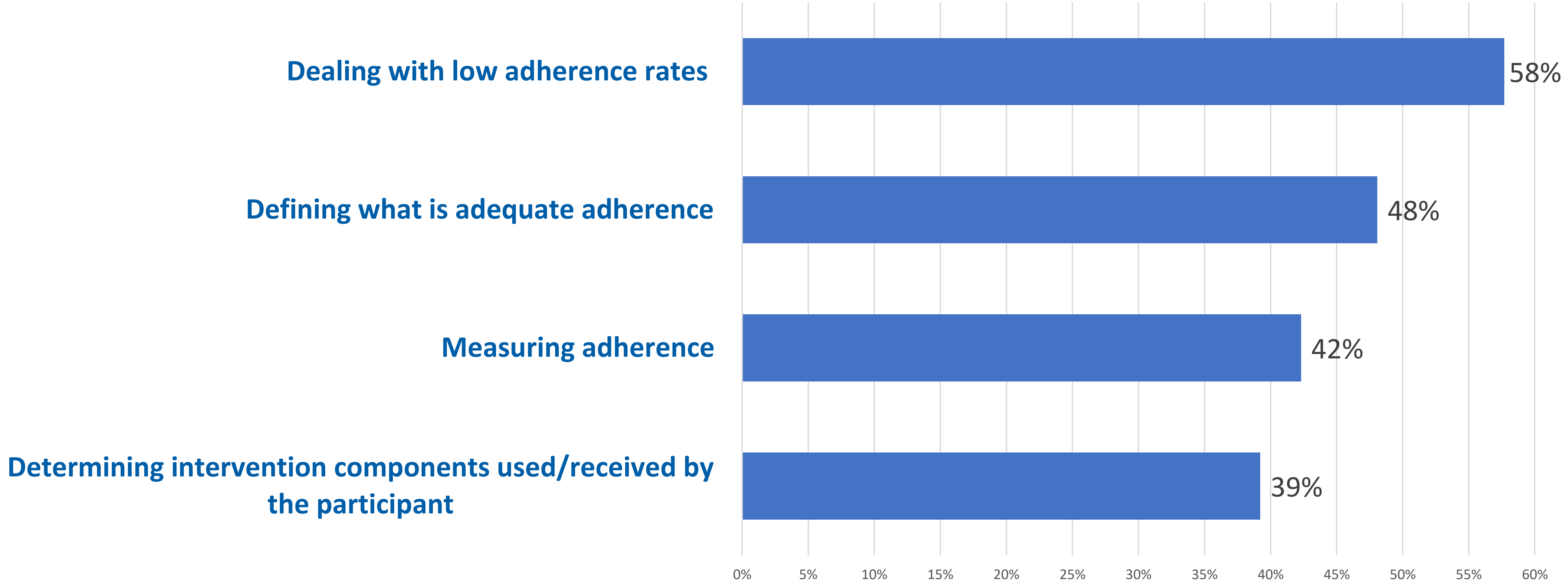

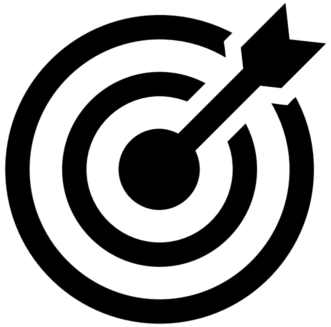

d. Workshop aim

To develop guidance to overcome methodological challenges related to **integrity** of mHealth interventions

Agenda

1 Feb 2023. 13:00 – 15:00 h CET

Cochrane Complementary Medicine

1	2	3	4
**Introduction of participants**	**Project presentation**	**Developing guidance**	**Closing remarks**
13:00 – 13:15	13:15 – 13:30	13:30 – 14:50	14:50 – 15:00
		Claudia M. Witt	L. Susan Wieland

USZ Universitäts Spital Zürich

Universität Zürich[UZH]

UNIVERSITY of MARYLAND SCHOOL OF MEDICINE

Topics: integrity of mHealth interventions

How did you deal with the following challenges?

1	How to measure adherence	42%
2	Defining what is adequate adherence	48%
3	Dealing with low adherence rates	58%
4	Determining intervention components used/received by the participant	39%
5	Dealing with apps that are continuously updated	Not surveyed

5. Closing remarks

Thank you for your support!

For more information:

Jesús López-Alcalde
Jesus.lopez@usz.ch

USZ Universitäts Spital Zürich

Universität Zürich[UZH]

UNIVERSITY of MARYLAND SCHOOL OF MEDICINE

Cochrane Complementary Medicine
