# Supplementary material for: Methodological Challenges in Randomized Controlled Trials of mHealth Interventions: Cross-Sectional Survey Study and Consensus-Based Recommendations
Source: J Med Internet Res. 2024 Dec 19;26:e53187. doi: 10.2196/53187 (PMC11695959; doi:10.2196/53187)
Supplement: Multimedia Appendix 6 [file jmir_v26i1e53187_app6.docx]

## Appendix 6. Additional challenges reported by the respondents (free text) in the survey

## I. Challenges in the recruitment

▪ Determining how to reward participation in mhealth RCTs

## II. Challenges in the randomization

▪ Conducting a single-arm mHealth study with a micro-randomized design.

## III. Challenges related to the intervention integrity

▪ Challenges with equipment shipping (e.g., delays) and loss (e.g., lost deliveries, equipment not being returned).

▪ Training participants to set up and use technology remotely, such as setting up the Bluetooth scale and connecting it to their phone.

▪ Getting third-party technology to work when the trial team does not have control over its technical aspects.

▪ New versions/updates to operating systems or apps create unintended problems (syncing, *etc*.)

▪ Consistently finding greater participation in follow-up in control groups vs. intervention groups

▪ Contamination between study arms (mainly the potential for large proportions of the control group accessing the intervention).

▪ One device for multiple family users.

▪ Downloading the app on the participants' personal tablets, which are used for other issues, can provide enough distraction to alter the study results.

▪ The app's operating system (iOS vs. Android) can affect the app's performance and, thus, impact the study outcomes.

▪ Elementary app details can impact measures such as pain and anxiety, particularly in the placebo app group.

▪ Technical support by the study personnel may not be anticipated in extent (for example, helping participants delete unused apps on phones so they could upload ours)

▪ Dealing with continuously evolving apps that may not be available in 2-3 years as technology changes rapidly.

▪Difficulties in measuring the acceptance of the mhealth app.

## IV. Challenges related to data quality

No additional challenges were identified.

## V. Challenges in data analysis

▪For trial teams not intimately involved in the app's functionality, it can be challenging to assess the effects of the app design and the users' choices.

## VI. Other challenges not related to the previous topics

▪To find an adequate control condition is complex. Assessing whether a mHealth app is effective will be very difficult without a placebo app.

▪Building/choosing a placebo app is challenging: the slightest changes, even in the sequence of offerings, can change results.
