# Supplementary material for: Methodological Challenges in Randomized Controlled Trials of mHealth Interventions: Cross-Sectional Survey Study and Consensus-Based Recommendations
Source: J Med Internet Res. 2024 Dec 19;26:e53187. doi: 10.2196/53187 (PMC11695959; doi:10.2196/53187)
Supplement: Multimedia Appendix 7 [file jmir_v26i1e53187_app7.docx]

## Multimedia appendix 7. Checklist for Reporting Results of Internet E-Surveys (CHERRIES)

| ***Checklist Item*** | ***Explanation*** | ***Page Number*** |
| --- | --- | --- |
| Describe survey design | Describe target population, sample frame. Is the sample a convenience sample? (In “open” surveys this is most likely) | See Methods: Phase 1- Study design and survey sample |
| IRB approval | Mention whether the study has been approved by an IRB. | See Methods- Ethical considerations |
| Informed consent | Describe the informed consent process. Where were the participants told the length of time of the survey, which data were stored and where and for how long, who the investigator was, and the purpose of the study? | See Methods: Phase 1- survey sample and ethical considerations.  All these items were detailed in the invitation email but which data were stored and where and for how long. However, participants were encouraged to contact the research team with questions. |
| Data protection | If any personal information was collected or stored, describe what mechanisms were used to protect unauthorized access. | See Methods- Ethical considerations |
| Development and testing | State how the survey was developed, including whether the usability and technical functionality of the electronic questionnaire had been tested before fielding the questionnaire. | See Methods: Phase 1- survey items |
| Open survey versus closed survey | An “open survey” is a survey open for each visitor of a site, while a closed survey is only open to a sample which the investigator knows (password-protected survey). | See Methods: Phase 1- survey items |
| Contact mode | Indicate whether or not the initial contact with the potential participants was made on the Internet. (Investigators may also send out questionnaires by mail and allow for Web-based data entry.) | See Methods: Phase 1- survey sample |
| Advertising the survey | How/where was the survey announced or advertised? Some examples are offline media (newspapers), or online (mailing lists – If yes, which ones?) or banner ads (Where were these banner ads posted and what did they look like?). It is important to know the wording of the announcement as it will heavily influence who chooses to participate. Ideally the survey announcement should be published as an appendix. | See Methods: Phase 1- survey sample. The potential participants were invited via email. The invitation email is presented in the Supplementary Materials. |
| Web/E-mail | State the type of e-survey (eg, one posted on a Web site, or one sent out through e-mail). If it is an e-mail survey, were the responses entered manually into a database, or was there an automatic method for capturing responses? | See Methods: Phase 1- survey sample. The potential participants were invited via email. The invitation email is presented in the Supplementary Materials. |
| Context | Describe the Web site (for mailing list/newsgroup) in which the survey was posted. What is the Web site about, who is visiting it, what are visitors normally looking for? Discuss to what degree the content of the Web site could pre-select the sample or influence the results. For example, a survey about vaccination on a anti-immunization Web site will have different results from a Web survey conducted on a government Web site | Not applicable |
| Mandatory/voluntary | Was it a mandatory survey to be filled in by every visitor who wanted to enter the Web site, or was it a voluntary survey? | See Methods: Phase 1- survey items: voluntary |
| Incentives | Were any incentives offered (eg, monetary, prizes, or non-monetary incentives such as an offer to provide the survey results)? | See Methods: Phase 1- survey sample: No incentives were offered to participate in the survey |
| Time/Date | In what timeframe were the data collected? | See Methods: Phase 1- survey items: “The survey was available from September 23 to November 22, 2022” |
| Randomization of items or questionnaires | To prevent biases items can be randomized or alternated. | See Methods: Phase 1- survey items: “The survey items were not randomized or alternated.” |
| Adaptive questioning | Use adaptive questioning (certain items, or only conditionally displayed based on responses to other items) to reduce number and complexity of the questions. | See Methods: Phase 1- survey items: “Adaptive questioning (certain items were conditionally displayed based on responses to other items) was implemented to reduce number of items.” |
| Number of Items | What was the number of questionnaire items per page? The number of items is an important factor for the completion rate. | See Methods: Phase 1- survey items: “The questionnaire consisted of a total of 29 questions distributed over six pages with a maximum of 21 questions per page” |
| Number of screens (pages) | Over how many pages was the questionnaire distributed? The number of items is an important factor for the completion rate. | See Methods: Phase 1- survey items: “The questionnaire consisted of 29 questions distributed over six pages with a maximum of six questions per page” |
| Completeness check | It is technically possible to do consistency or completeness checks before the questionnaire is submitted. Was this done, and if “yes”, how (usually JAVAScript)? An alternative is to check for completeness after the questionnaire has been submitted (and highlight mandatory items). If this has been done, it should be reported.  All items should provide a non-response option such as “not applicable” or “rather not say”, and selection of one response option should be enforced. | See Methods: Phase 1- survey items: “After each section of the questionnaire was filled-in, participants had the opportunity to check for completeness. If any items were left unanswered, a message would appear to highlight the missing responses. Completing the missing items was not mandatory to minimize survey fatigue.”  See Methods: Phase 1- survey items: “An "I don't know" option was also available.” |
| Review step | State whether respondents were able to review and change their answers (eg, through a Back button or a Review step which displays a summary of the responses and asks the respondents if they are correct). | See Methods: Phase 1- survey items: “Respondents were able to review and change their answers by clicking the back button”. |
| Unique site visitor | If you provide view rates or participation rates, you need to define how you determined a unique visitor. There are different techniques available, based on IP addresses or cookies or both. | Not applicable, we do not provide view or participation rates. We have explained this in Survey sample: "Participants could complete the survey multiple times, as the survey platform did not restrict submissions to unique visitors." |
| View rate (Ratio of unique survey visitors/unique site visitors) | Requires counting unique visitors to the first page of the survey, divided by the number of unique site visitors (not page views!). It is not unusual to have view rates of less than 0.1 % if the survey is voluntary. | Not applicable, we do not have the number of unique visitors |
| Participation rate (Ratio of unique visitors who agreed to participate/unique first survey page visitors) | Count the unique number of people who filled in the first survey page (or agreed to participate, for example by checking a checkbox), divided by visitors who visit the first page of the survey (or the informed consents page, if present). This can also be called “recruitment” rate. | Not applicable, we do not have the number of unique visitors |
| Completion rate (Ratio of users who finished the survey/users who agreed to participate) | The number of people submitting the last questionnaire page, divided by the number of people who agreed to participate (or submitted the first survey page). This is only relevant if there is a separate “informed consent” page or if the survey goes over several pages. This is a measure for attrition. Note that “completion” can involve leaving questionnaire items blank. This is not a measure for how completely questionnaires were filled in. (If you need a measure for this, use the word “completeness rate”.) | See survey results: The completion rate was 40.2% (number of people who agreed to participate (n=106) divided by number of visitors (n=264)) |
| Cookies used | Indicate whether cookies were used to assign a unique user identifier to each client computer. If so, mention the page on which the cookie was set and read, and how long the cookie was valid. Were duplicate entries avoided by preventing users access to the survey twice; or were duplicate database entries having the same user ID eliminated before analysis? In the latter case, which entries were kept for analysis (eg, the first entry or the most recent)? | See survey sample: “Cookies were not used to assign a unique user identifier to each client computer. Additionally, IP addresses were not utilized to detect potential duplicate entries from the same user, and no other methods were employed to analyze the log file for identifying multiple submissions." |
| IP check | Indicate whether the IP address of the client computer was used to identify potential duplicate entries from the same user. If so, mention the period of time for which no two entries from the same IP address were allowed (eg, 24 hours). Were duplicate entries avoided by preventing users with the same IP address access to the survey twice; or were duplicate database entries having the same IP address within a given period of time eliminated before analysis? If the latter, which entries were kept for analysis (eg, the first entry or the most recent)? | See survey sample: “Cookies were not used to assign a unique user identifier to each client computer. Additionally, IP addresses were not utilized to detect potential duplicate entries from the same user, and no other methods were employed to analyze the log file for identifying multiple submissions." |
| Log file analysis | Indicate whether other techniques to analyze the log file for identification of multiple entries were used. If so, please describe. | See survey sample: “Cookies were not used to assign a unique user identifier to each client computer. Additionally, IP addresses were not utilized to detect potential duplicate entries from the same user, and no other methods were employed to analyze the log file for identifying multiple submissions." |
| Registration | In “closed” (non-open) surveys, users need to login first and it is easier to prevent duplicate entries from the same user. Describe how this was done. For example, was the survey never displayed a second time once the user had filled it in, or was the username stored together with the survey results and later eliminated? If the latter, which entries were kept for analysis (eg, the first entry or the most recent)? | Not applicable, the survey was open. |
| Handling of incomplete questionnaires | Were only completed questionnaires analyzed? Were questionnaires which terminated early (where, for example, users did not go through all questionnaire pages) also analyzed? | See Survey analysis: “Individuals who consented to participate but rated less than 50% of the listed challenges were excluded. Subjects responding "I don't know" or not responding to a particular item were excluded from the analysis for that item” |
| Questionnaires submitted with an atypical timestamp | Some investigators may measure the time people needed to fill in a questionnaire and exclude questionnaires that were submitted too soon. Specify the timeframe that was used as a cut-off point, and describe how this point was determined. | See Survey analysis: “There was no time limit for completing the questionnaire in order for the responses to be included.” |
| Statistical correction | Indicate whether any methods such as weighting of items or propensity scores have been used to adjust for the non-representative sample; if so, please describe the methods. | See Survey analysis: “There were no statistical corrections to adjust for the non-representative sample” |

This checklist has been modified from Eysenbach G. Improving the quality of Web surveys: the Checklist for Reporting Results of Internet E-Surveys (CHERRIES). J Med Internet Res. 2004 Sep 29;6(3):e34 [erratum in J Med Internet Res. 2012; 14(1): e8.]. Article available at [https://www.jmir.org/2004/3/e34](https://www.jmir.org/2004/3/e34/)/; erratum available <https://www.jmir.org/2012/1/e8/>. Copyright ©Gunther Eysenbach. Originally published in the [Journal of Medical Internet](http://www.jmir.org) Research, 29.9.2004 and 04.01.2012.

This is an open-access article distributed under the terms of the Creative Commons Attribution License (<https://creativecommons.org/licenses/by/2.0/>), which permits unrestricted use, distribution, and reproduction in any medium, provided the original work, first published in the Journal of Medical Internet Research, is properly cited.
