# Supplementary material for: Methodological Challenges in Randomized Controlled Trials of mHealth Interventions: Cross-Sectional Survey Study and Consensus-Based Recommendations
Source: J Med Internet Res. 2024 Dec 19;26:e53187. doi: 10.2196/53187 (PMC11695959; doi:10.2196/53187)
Supplement: Multimedia Appendix 8 [file jmir_v26i1e53187_app8.docx]

## Multimedia Appendix 8: Checklist for Accurate Consensus Reporting Document guidelines

| Item No. | Section | Checklist Item (*help text*) | Page No. |
| --- | --- | --- | --- |
| T1 | **Title** | Identify the article as reporting a consensus exercise and state the consensus methods used in the title.  *For example, Delphi or nominal group technique.* | Done: “Methodological challenges in randomized controlled trials of mHealth interventions: survey and consensus-based recommendations”  Including the consensus methods in the title is unnecessary, as this project was a mixed-methods study that did not solely focus on consensus. |
| I1 | **Introduction** | Explain why a consensus exercise was chosen over other approaches. | See introduction: “A survey and a consensus exercise were chosen for this project to address the diverse methodological challenges in mHealth RCTs. Consensus methods allow for the integration of expert perspectives to produce recommendations. This approach is essential given the lack of consistent solutions to these challenges.” |
| I2 |  | State the aim of the consensus exercise, including its intended audience and geographical scope (national, regional, global). | See Introduction: “The goal of the consensus exercise was to develop recommendations for researchers working in mHealth. The intended audience is global, with participation from experts across regions, making the recommendations relevant for both high- and low-resource settings.” |
| I3 |  | If the consensus exercise is an update of an existing document, state why an update is needed, and provide the citation for the original document. | Not applicable |
| M1 | **Methods**  Registration | If the study or study protocol was prospectively registered, state the registration platform and provide a link. If the exercise was not registered, this should be stated.  *Recommended to include the date of registration.* | See Methods: “The study protocol was not registered.” |
| M2 | Selection of SC and/or panellists | Describe the role(s) and areas of expertise or experience of those directing the consensus exercise.  *For example, whether the project was led by a chair, co-chairs or a steering committee, and, if so, how they were chosen. List their names if appropriate, and whether there were any subgroups for individual steps in the process.* | See Methods: “The steering group, which included four researchers (Claudia Witt (CW), Lisa Susan Wieland (LSW), Jürgen Barth (JB) and Jesús López-Alcalde (JLA)), defined the project aims, collected potential methodological challenges of mHealth RCTs, identified the potential survey respondents, developed the survey, and selected the workshop participants. JB, an experienced researcher, facilitated the consensus meeting. The steering group also analyzed the survey results, summarized participants' comments during the workshop, and integrated their feedback to generate this manuscript. No members of the public, patients or carers were invited to participate in the study.” |
| M3 |  | Explain the criteria for panellist inclusion and the rationale for panellist numbers. State who was responsible for panellist selection. | See Methods and Results: ·”We invited the 52 survey respondents who indicated their interest in participating in a workshop to develop consensus-based recommendations addressing methodological challenges specific to mHealth RCTs” […] “Eleven mHealth researchers attended the online workshop” |
| M4 |  | Describe the recruitment process (how panellists were invited to participate).  *Include communication/advertisement method(s) and locations, numbers of invitations sent, and whether there was centralised oversight of invitations or if panellists were asked/allowed to suggest other members of the panel.* | See above |
| M5 |  | Describe the role of any members of the public, patients or carers in the different steps of the study. | See Methods: “No members of the public, patients or carers were invited to participate in the study” |
| M6 | Preparatory research | Describe how information was obtained prior to generating items or other materials used during the consensus exercise.  *This might include a literature review, interviews, surveys, or another process.* | See Methods, which describes how the survey items were collated and how we collected the data on those items |
| M7 |  | Describe any systematic literature search in detail, including the search strategy and dates of search or the citation if published already.  *Provide the details suggested by the reporting guideline PRISMA and the related PRISMA-Search extension.* | This information is detailed in Appendix 1. |
| M8 |  | Describe how any existing scientific evidence was summarised and if this evidence was provided to the panellists. | Not applicable |
| M9 | Assessing consensus | Describe the methods used and steps taken to gather panellist input and reach consensus (for example, Delphi, RAND-UCLA, nominal group technique).  *If modifications were made to the method in its original form, provide a detailed explanation of how the method was adjusted and why this was necessary for the purpose of your consensus-based study.* | See Methods: “The consensus method involved a consensus meeting, wherein participants engaged in discussions to reach an agreement. Recommendations from the workshop were endorsed by consensus without structured voting” |
| M10 |  | Describe how each question or statement was presented and the response options. State whether panellists were able to or required to explain their responses, and whether they could propose new items.  *Where possible, present the questionnaire or list of statements as supplementary material.* | See Methods |
| M11 |  | State the objective of each consensus step.  *A step could be a consensus meeting, a discussion or interview session, or a Delphi round.* | See Methods |
| M12 |  | State the definition of consensus (for example, number, percentage, or categorical rating, such as ‘agree’ or ‘strongly agree’) and explain the rationale for that definition. | See Methods: “The consensus method involved a consensus meeting, wherein participants engaged in discussions to reach an agreement. Recommendations from the workshop were endorsed by consensus without structured voting [26]. See Appendix 4: Workshop slides.” |
| M13 |  | State whether items that met the prespecified definition of consensus were included in any subsequent voting rounds. | Not applicable |
| M14 |  | For each step, describe how responses were collected, and whether responses were collected in a group setting or individually. | Not applicable |
| M15 |  | Describe how responses were processed and/or synthesised.  *Include qualitative analyses of free-text responses (for example, thematic, content or cluster analysis) and/or quantitative analytical methods, if used.* | See methods: “JLA and CW performed a thematic analysis to summarise the workshop discussions”· |
| M16 |  | Describe any piloting of the study materials and/or survey instruments.  *Include how many individuals piloted the study materials, the rationale for the selection of those individuals, any changes made as a result and whether their responses were used in the calculation of the final consensus. If no pilot was conducted, this should be stated.* | See Methods: “The usability and technical functionality of the electronic questionnaire was tested by the steering group and two external researchers before fielding the questionnaire”. |
| M17 |  | If applicable, describe how feedback was provided to panellists at the end of each consensus step or meeting.  *State whether feedback was quantitative (for example, approval rates per topic/item) and/or qualitative (for example, comments, or lists of approved items), and whether it was anonymised.* | See Methods: “The survey results and the workshop recommendations were integrated into the first manuscript draft, which was emailed to the workshop participants. JLA incorporated their feedback into the final version of the manuscript” |
| M18 |  | State whether anonymity was planned in the study design. Explain where and to whom it was applied and what methods were used to guarantee anonymity. | See Methods: “We conducted an anonymous online survey using the SoSci Survey platform, hosted by University Hospital Zurich. In the final section of the survey, respondents were invited to participate in an optional online workshop” |
| M19 |  | State if the steering committee was involved in the decisions made by the consensus panel.  *For example, whether the steering committee or those managing consensus also had voting rights.* | See Methods: “JLA and CW performed a thematic analysis to summarise the workshop discussions [29], see steps in figure 1.” |
| M20 | Participation | Describe any incentives used to encourage responses or participation in the consensus process.  *For example, were invitations to participate reiterated, or were participants reimbursed for their time.* | See Methods: “No incentives were offered to participate in the survey. […] While workshop participants were not offered financial compensation, they were given the opportunity to be co-authors of the manuscript if they met the authorship criteria.” |
| M21 |  | Describe any adaptations to make the surveys/meetings more accessible.  *For example, the languages in which the surveys/meetings were conducted and whether translations or plain language summaries were available*. | See Methods. The survey was available in English for two months. The usability and technical functionality of the electronic questionnaire was tested by the steering group and two external researchers before fielding the questionnaire. The online workshop was in English |
| R1 | Results | State when the consensus exercise was conducted. List the date of initiation and the time taken to complete each consensus step, analysis, and any extensions or delays in the analysis. | See Results: “Eleven mHealth researchers attended the online workshop (2 hours, 1 February 2023) (Table 3).” |
| R2 |  | Explain any deviations from the study protocol, and why these were necessary.  *For example, addition of panel members during the exercise, number of consensus steps, stopping criteria; report the step(s) in which this occurred.* | Not applicable |
| R3 |  | For each step, report quantitative (number of panellists, response rate) and qualitative (relevant socio-demographics) data to describe the participating panellists. | See results |
| R4 |  | Report the final outcome of the consensus process as qualitative (for example, aggregated themes from comments) and/or quantitative (for example, summary statistics, score means, medians and/or ranges) data. | See results: “The following recommendations were agreed upon during the workshop and refined by email afterwards following a qualitative process” |
| R5 |  | List any items or topics that were modified or removed during the consensus process. Include why and when in the process they were modified or removed. | Not applicable |
| D1 | Discussion | Discuss the methodological strengths and limitations of the consensus exercise.  *Include factors that may have impacted the decisions (for example, response rates, representativeness of the panel, potential for feedback during consensus to bias responses, potential impact of any non-anonymised interactions).* | See discussion |
| D2 |  | Discuss whether the recommendations are consistent with any pre-existing literature and, if not, propose reasons why this process may have arrived at alternative conclusions. | See “Comparison with Prior Work” |
| O1 | Other information | List any endorsing organisations involved and their role. | Not applicable |
| O2 |  | State any potential conflicts of interests, including among those directing the consensus study and panellists. Describe how conflicts of interest were managed. | See “Funding” and “Conflicts of Interest” |
| O3 |  | State any funding received and the role of the funder.  *Specify, for example, any funder involvement in the study concept/design, participation in the steering committee, conducting the consensus process, funding of any medical writing support. This could be disclosed in the methods or in the relevant transparency section of the manuscript. Where a funder did not play a role in the process or influence the decisions reached, this should be specified.* | See “Funding” and “Conflicts of Interest” |

For more information see: https://www.ismpp.org/accord
